# Pan-European analysis shows stable, low antimicrobial resistance in most bovine and porcine respiratory tract pathogens

**DOI:** 10.3389/fmicb.2026.1745115

**Published:** 2026-02-23

**Authors:** Anno de Jong, Robin Temmerman, Markus Rose, Shabbir Simjee, Farid El Garch, on behalf of the VetPath Study Group

**Affiliations:** VetPath Study Group, CEESA, Brussels, Belgium

**Keywords:** antimicrobial resistance surveillance, cattle, minimal inhibitory concentrations, pigs, respiratory infections

## Abstract

**Aims:**

To survey antibiotic susceptibility of bacteria causing bovine and porcine respiratory tract infections in eight European countries and to compare retrospectively the resistance percentages between the countries participating in the VetPath programs.

**Methods and results:**

Non-replicate nasopharyngeal/nasal or lung swabs were collected by harmonized sampling from animals with acute respiratory signs during the period 2019–2020. *Pasteurella multocida*, *Mannheimia haemolytica*, *Histophilus somni* from cattle (*n* = 307), and *P. multocida*, *Actinobacillus pleuropneumoniae*, *Glaesserella parasuis*, *Bordetella bronchiseptica* and *Streptococcus suis* from pigs (*n* = 659) were isolated by standard methods. *S. suis* was also isolated from meningitis cases. Minimum Inhibitory Concentrations (MICs) were assessed following CLSI standards and interpreted using veterinary breakpoints where available. *H. somni* isolates were fully susceptible against all tested antibiotics. Resistance in bovine *P. multocida* and *M. haemolytica* was absent or low, except for tetracycline (29.7 and 19.6% resistance for the two bacteria, respectively). Low macrolide and spectinomycin resistance were observed for *M. haemolytica* (1.5–2.3%) contrary to *P. multocida* (8.3–22.1%). Similar susceptibility patterns were observed in pigs. Resistance in *P. multocida*, *A. pleuropneumoniae* and *S. suis* to ceftiofur, enrofloxacin, florfenicol, and penicillin was absent or <3.0%. Tetracycline resistance varied from 11.4 to 13.9% but was 83.2% in *S. suis*. For all pathogens multidrug-resistance was low (0–6.2%). Overall, antibiotic resistance in the 2019–2020 survey remained similar as in the 2009–2012 and 2015–2016 surveys. Among the countries significant variations of resistance levels were observed, particularly for tetracycline. Drivers behind these country differences remain unclear.

**Conclusion:**

With the exception of tetracycline, low antibiotic resistance was observed among major bovine and porcine respiratory tract pathogens in Europe.

## Introduction

Antimicrobial resistance (AMR) has become one of the greatest challenges in modern medicine, posing threats to both human and animal populations. It is on many national and international political agendas (World Health Organization, 2015; European Commission, 2017; World Organisation for Animal Health, 2021; Centre for Disease Prevention and Control, 2022). While antimicrobial agents once seemed to be the solution for treatment of infectious diseases, today’s reality is quite different, with almost all pathogenic bacteria showing resistance traits against antimicrobial agents. AMR not only concerns strains affecting human health but also threatens sustainable food production and environmental health, requiring coordinated global efforts to address it. Hence, a One Health approach is required to combat the spread of antimicrobial-resistant organisms in animals, the environment, and in humans in the community and hospital settings (Hernando-Amado et al., 2019; Martak et al., 2024; ECDC, EFSA, EMA, 2024; World Health Organization, 2024). The One Health approach emphasizes the standardization of surveillance methods for AMR across nations, and encourages countries to implement domestic action plans to monitor and to assess AMR patterns of clinical isolates (Boqvist et al., 2018; Ma et al., 2021; World Health Organization, 2025). Although AMR-associated foodborne and zoonotic bacterial pathogens are known to transfer between animals and humans, it remains unclear whether the use of antimicrobials in farm animals directly drives AMR in humans (Tang et al., 2017; Martak et al., 2022). The importance of validated, harmonized and continuous monitoring of AMR of veterinary pathogens as a component of good stewardship has been frequently highlighted (Schwarz et al., 2010; Feßler et al., 2023; Krüger-Haker et al., 2026). As increasing AMR has been reported in the past decades, antimicrobial susceptibility should be carefully monitored to identify trends over time to ensure long-term efficacy of the antibacterial products.

Respiratory diseases are a significant cause of morbidity and mortality among livestock resulting in reduced animal welfare and high economic losses in farming. Hence, various management measures to prevent or to treat bovine respiratory disease (BRD) and swine respiratory disease (SRD) are important, including the use of vaccines and antibiotics. Many antimicrobial products have indications against the most commonly diagnosed pathogens causing pneumonia in cattle and pigs, which include members of the *Pasteurellaceae* family: *Pasteurella multocida*, *Mannheimia haemolytica*, *Histophilus somni*, *Actinobacillus pleuropneumoniae* and *Glaesserella parasuis* (Woolums, 2015; Zimmerman et al., 2019; Wiencek et al., 2022; Apply et al., 2024). *Bordetella bronchiseptica* is another organism involved in the etiology of bronchopneumonia (and atrophic rhinitis) in pigs and is also a contributing agent in the multifactorial porcine respiratory disease complex (Kadlec and Schwarz, 2018; Zhang et al., 2021). *B. bronchiseptica* infections are often accompanied by co-infection with other bacteria such as *P. multocida* or *G. parasuis* (Kadlec et al., 2004)*. Streptococcus suis*, another major respiratory tract pathogen responsible for pneumonia in pigs, can also cause a variety of other diseases including meningitis, arthritis, peri- and endocarditis, polyserositis, septicemia, abortion and sudden death (Segura et al., 2020; Dechêne-Tempier et al., 2021; Vilaró et al., 2025). Some pathogenic *S. suis* strains have zoonotic potential, causing meningitis in humans (Weinert et al., 2015).

In Europe, the veterinary pharmaceutical industry has conducted periodic monitoring programs of the antimicrobial susceptibility of veterinary pathogens from food animals through the Executive Animal Health Study Centre (CEESA) (De Jong et al., 2013). These programs are based on harmonized methods of sampling and bacterial isolation to establish collections of representative pathogens from diseased livestock animals. There are also several valuable national programs, e.g., GE*RM-*Vet in Germany or Resapath in France, which are systematically monitoring AMR in their respective countries (GE*RM*-Vet, 2022; Anses, 2024), additionally, one-off studies frequently report susceptibility data (Van Hout et al., 2016; Vilaró et al., 2020; Kudirkiene et al., 2021; Hennig-Pauka et al., 2022). Recently an initiative to establish an extended European AMR surveillance network designated EARS-Vet was made (Mader et al., 2021). This proposal is similar to initiatives already in place in human medicine, where the European Centre for Disease Prevention and Control (ECDC) coordinates the European Antimicrobial Resistance Surveillance Network (EARS-Net), which monitors AMR in invasive bacteria in hospitalized patients in a harmonized way (European Centre for Disease Prevention and Control, 2019). EARS-Vet proposes that the European national surveys monitor AMR in a fully standardized manner in four food animal species (and in dogs and cats) for 10 bacterial species (Mader et al., 2022). A similar initiative has been taken by Teale and Borriello (2021), who have defined several bacterial species, antimicrobial agents and dilution ranges covering where possible both clinical breakpoints and epidemiological cut-off values (ECOFFs). However, so far these AMR initiatives have recently started and require more time to be developed. Moreover, they are dependent on political commitment and sustained funding (Mader et al., 2021).

VetPath, the CEESA pan-European antimicrobial susceptibility monitoring program for target pathogens from food-producing animals, has recently reported data on BRD and SRD pathogens in various European countries (El Garch et al., 2016, 2020). The current paper presents the findings for eight major respiratory tract pathogens associated with BRD or SRD for 2019–2020. However, low numbers of isolates per country have precluded a comparison of resistance percentages across countries in the individual VetPath studies. In the current study, therefore, data of the VetPath studies 2009–2012, 2015–2016 and 2019–2020 are retrospectively combined and analyzed to compare individual countries based on relevant number of isolates per country.

## Materials and methods

### Animal criteria and sampling procedures

In each country, samples were only taken from diseased or recently deceased animals with acute clinical signs of respiratory tract infections, and in pigs additionally from meningitis samples for recovery of *S. suis*, and these animals were supposed to have not been exposed to antibacterial treatment for at least 15 days prior to sampling. Overall, 60% of the samples came from confirmed recently untreated livestock and 40% of the samples were from animals with the pre-treatment status “unknown”. One of the principles of our monitoring program is to collect samples from cattle and pigs not recently exposed to antibiotic treatments in order to avoid biased results in favor of resistance. Antimicrobial susceptibility data from recently treated animals might be not representative for isolates from patients without a history of antimicrobial therapy, and might overestimate the occurrence of resistance. However, analysis of the percentages of resistance of both populations in preceding VetPath studies failed to find differences between the two populations. Pooled results, therefore, are being presented in this paper. In order to reflect the reality of the field conditions, only bacterial strains from animals suffering from acute clinical infections have been included; chronically diseased animals were excluded. Responsibility for the provision of the clinical samples and processing according to uniform protocols for pathogen isolation was allocated to a single national coordinator based in each participating country. The national coordinators strictly followed the protocol. The coordinators arranged that the samples from clinical or post mortem cases were sent in appropriate conditions directly to the approved regional veterinary diagnostic laboratories and stored until recovery of the bacteria. Details of additional criteria and sampling procedures have been described elsewhere (El Garch et al., 2016). For each sample, all relevant data were provided on a standard case report form although sometimes the antibiotic pretreatment data were not provided despite investigators being aware that this was crucial information. Only one isolate per outbreak per farm was retained for further characterization, in order to obtain a collection of epidemiologically unrelated strains. All isolates from pre-treated animals were excluded from the collection. In order to have a valid data set representative for Europe, the participating laboratories of each country were given minimum and maximum target numbers for submission of isolates. Owing to the very strict inclusion criteria, it was not always possible to achieve the target number of isolates in each country.

Each eight countries were selected for cattle (Belgium, Czech Republic, France, Germany, Italy, Spain, The Netherlands, the United Kingdom) and pigs (Belgium, Denmark, France, Germany, Poland, Spain, The Netherlands, the United Kingdom). At maximum, each farm was only included once per 3 months (cattle) or per 6 months (pigs) in the survey. The isolates were identified to genus and species level by standard biochemical tests before shipment to a central laboratory (IHMA Europe Sàrl, Monthey, Switzerland). Briefly, nasal swabs, deep nasopharyngeal swabs, or bronchoalveolar lavage or lung samples (from euthanized or less than 24 h dead animal) from diseased animals were submitted to veterinary diagnostic laboratories for bacterial identification. First, microscopic examination using of cytology and Gram staining was performed to provide some clues on a suspicious pathogen. Then, inoculation onto culture media was performed. Usually, laboratories used combinations of non-selective (blood agar plate) and selective (MacConkey agar) media appropriate for Gram-positive or Gram-negative veterinary pathogens. Common respiratory targets included Gram-negative bacteria (*P. multocida, M. haemolytica*, *H. somni*, *A. pleuropneumoniae, B. bronchiseptica, G. parasuis*) and Gram-positive bacteria (*S. suis*). Once inoculated agar plates were incubated for 24–48 h and incubation parameters differed by organism type (aerobic +/− 5% CO_2_), according to the national diagnostic laboratories and practices. After incubation, plates were inspected for colony morphology, hemolysis patterns, odor, or growth on selective media. Identification of bacterial isolates was performed by combination of biochemical testing (commercial (i.e., API strips) or manual biochemical profiling), mass spectrometry (Matrix-Assisted Laser Desorption Ionization-Time of Flight Mass Spectrometry; MALDI-ToF MS), or molecular diagnostics (i.e., PCR). All isolates were re-identified by MALDI-ToF MS on Microflex LT (Bruker Daltonics, Bremen, Germany) at the central laboratory prior to antimicrobial susceptibility testing.

### Antimicrobial susceptibility testing

The central laboratory has determined the susceptibility as minimal inhibitory concentration (MIC) using a panel of licensed antimicrobial agents commonly used for the treatment of respiratory tract infections in cattle and pigs in Europe. The antimicrobial classes that are approved in Europe are beta-lactam antibiotics, fluoroquinolones, phenicols, tetracyclines, trimethoprim/sulfonamides, aminoglycosides/aminocyclitols, lincosamides, macrolides, and pleuromutilins. MICs for the isolates were determined using a broth microdilution method according to Clinical and Laboratory Standard Institute microtitre plates containing a total of 24 antibiotics/antibiotic combinations (Clinical and Laboratory Standards Institute, 2018). The exception was the MIC testing of *G. parasuis*, where antimicrobial susceptibility was assessed in cation-adjusted Mueller Hinton broth supplemented with 0.0025% NADH and 1% sterile heat-inactivated chicken serum (Prüller et al., 2017). Details of the susceptibility testing have been described elsewhere (El Garch et al., 2016). Quality control strains as specified by CLSI were included in all testing runs (Clinical and Laboratory Standards Institute, 2018). The selected antibiotics and their concentration ranges are included in Supplementary Tables S4–S11. All ranges were chosen to encompass appropriate quality control ranges and veterinary clinical breakpoints (Clinical and Laboratory Standards Institute, 2024). The MICs of the reference strains in each test run were within the CLSI quality control ranges.

### Data analysis

The MIC_50_ and MIC_90_ values were determined for each antibiotic. Where CLSI veterinary breakpoints were available (Clinical and Laboratory Standards Institute, 2024), the percentage susceptible, intermediate and resistant isolates was reported. Breakpoints are indicated by vertical bars in the MIC distribution tables (Supplementary Tables S4–S11). If no species-specific interpretive criteria were available for an antimicrobial, a classification in percentage susceptible, intermediate and resistant isolates was not possible, which is indicated with a dash in Supplementary Tables S4–S11.

The occurrence of multidrug-resistance (MDR) was determined according to the definition set by Sweeney et al. (2018): Isolates are classified as MDR if they are resistant to at least one agent in three or more antimicrobial class categories. Following this definition, the MDR was determined in this study for *P. multocida*, *M. haemolytica*, *H. somni* from cattle and for *P. multocida*, *A. pleuropneumoniae*, *B. bronchiseptica* and *S. suis* from pigs. As there are no species-specific breakpoints available for *G. parasuis,* the calculation of MDR is precluded. The antibiotics included were seven of the most commonly used antimicrobials for the cattle pathogens: ceftiofur, enrofloxacin (representative for the fluoroquinolones), florfenicol, penicillin G, spectinomycin, tetracycline and tulathromycin (representative for the macrolide antibiotics) and five to seven antibiotics for the pig pathogens: ceftiofur, enrofloxacin, florfenicol, penicillin G (only *P. multocida*, *S. suis*), tetracycline, tiamulin (only *A. pleuropneumoniae*) and tulathromycin (only *P. multocida*, *A. pleuropneumoniae*).

Percentages of resistance of VetPath 2019–2020 were compared with the values observed in the preceding sampling periods of VetPath (2009–2012 and 2015–2016) by the non-parametric Mann–Whitney test (two-sided) for the six major pathogens for eight antibiotics tested. For all tests, a *p* value of ≤ 0.05 was considered as a significant difference.

A comparison of the resistance levels in individual countries was performed retrospectively for the combined time periods 2009–2012 (El Garch et al., 2016), 2015–2016 (de Jong et al., 2023) and the current bacteria collection 2019–2020. This was conducted for all antimicrobial agents with defined breakpoints, for the major cattle (*P. multocida*, *M. haemolytica*) and pig pathogens (*P. multocida*, *A. pleuropneumoniae*, *S. suis*). Countries with less than 20 isolates from the entire study period were excluded from analysis. Two-sided *χ*^2^ tests were used for each pathogen for an overall comparison of the countries. In the case of a significant difference, pairwise comparisons of resistance prevalence between countries were used for each bacterial species and for each antimicrobial. A *p* value of ≤ 0.05 was considered significant (no control of multilevel alpha).

## Results

### Cattle isolates

In cattle, a total of 307 isolates were recovered from BRD cases: 145 *P. multocida,* 133 *M. haemolytica* and 29 *H. somni* (Supplementary Tables S4–S6). The large majority of these were susceptible to antibiotics for which a CLSI veterinary breakpoint is available. Resistance to ceftiofur was absent in all three organisms. For amoxicillin and cefquinome, no CLSI breakpoints are available. Amoxicillin shows a bimodal MIC distribution for *M. haemolytica*, suggesting the development of resistance in some isolates, whereas one *P. multocida* isolate had a distinctly higher MIC value. In contrast, MIC distribution for amoxicillin/clavulanic acid was monomodal, as applies to cefquinome and ceftiofur. The bovine isolates ranged in their colistin MICs between ≤0.06 and 16 μg mL^−1^ with MIC_50_ and MIC_90_ values of 0.25 and 0.25–2 μg mL^−1^, respectively. The MIC distributions for marbofloxacin were similar to that of enrofloxacin and danofloxacin, for which clinical resistance was low (0.8–2.8%). Similarly, resistance to florfenicol was low (2.1–2.3%) for the isolates from both species. The highest resistance percentage was observed for tetracycline at 29.7% for *P. multocida* and 19.6% for *M. haemolytica*, whereas resistance of *H. somni* was 3.5%. All *H. somni* isolates were susceptible to second-generation macrolides gamithromycin, tildipirosin and tulathromycin, whereas the resistance for the other two bovine pathogens varied between 1.5 and 11.0%. MIC values for the older macrolide tilmicosin were similar, whereas MICs for tylosin were markedly higher for *P. multocida* and *M. haemolytica*. For both species, MIC_90_ values were 16–128 μg mL^−1^ for tiamulin and lincomycin, while both compounds showed markedly lower MIC_90_ values for *H. somni* (0.5 and ≤4 μg mL^−1^, respectively). Lincomycin/spectinomycin and trimethoprim/sulfamethoxazole exhibited broad multimodal MIC patterns. While *H. somni* and *M. haemolytica* isolates exhibited low resistance to spectinomycin, 22.1% resistance was observed for *P. multocida*.

### Pig isolates

In total 659 isolates were recovered from SRD cases: 149 *P. multocida,* 151 *A. pleuropneumoniae,* 37 *G. parasuis,* 90 *B. bronchiseptica* from respiratory infections and 232 *S. suis* from respiratory tract infections (79 isolates) or meningitis infections (153 isolates) (Supplementary Tables S7–S11). With respect to the three primary species *P. multocida*, *A. pleuropneumoniae* and *S. suis,* resistance to the antibiotics tested was zero to low (0.0–5.3%), except for tetracycline where 11.4% of *P. multocida,* 13.9% of *A. pleuropneumoniae* and 83.2% of *S. suis* were resistant, and tilmicosin (11.3% resistant for *A. pleuropneumoniae*). Similarly, the percentage of isolates classified as intermediate was 0.0–7.3% for *P. multocida, A. pleuropneumoniae* and *S. suis,* except for tetracycline (13.4, 2.0 and 12.1% resistance, respectively). Few *Pasteurella*, *Actinobacillus* and *S. suis* isolates were out of the wild-type distribution for amoxicillin. Remarkably, for these three species the amoxicillin/clavulanic acid MICs showed a narrower range than for amoxicillin. MIC distributions of cefquinome and ceftiofur were similar, and resistance to ceftiofur was only observed for four *S. suis* isolates (1.7% resistance) (no CLSI breakpoints available for cefquinome). The colistin MIC distributions were primarily monomodal for *P. multocida* and *A. pleuropneumoniae*; for *S. suis* very high colistin MIC values exceeding 32 μg mL^−1^ were observed, which is in accordance with the intrinsic resistance of *S. suis* to colistin. MIC values for the three fluoroquinolones were similarly distributed. Almost all (>96.0%) *P. multocida,* and *A. pleuropneumoniae* isolates were susceptible to tulathromycin and tildipirosin. Low levels of tilmicosin resistance were observed for *P. multocida* (0.7%) and a moderate level for *A. pleuropneumoniae* (11.3%). For *S. suis*, the MIC distributions for tilmicosin, tylosin, and lincomycin were clearly bimodal, indicating acquired resistance among the isolates; the separation between the susceptible and resistant population was absent or less clear for *P. multocida* and *A. pleuropneumoniae*. Resistance to tiamulin amounted to 2.0% in *A. pleuropneumoniae;* the only species where a breakpoint for tiamulin has been set. Trimethoprim/sulfamethoxazole exhibited very wide, multimodal MIC distributions. Several isolates of *P. multocida* and *S. suis* had distinctly higher MICs to spectinomycin (>512 μg mL^−1^), indicating acquired resistance to this compound.

Among the five porcine pathogens, MICs of *B. bronchiseptica* were the highest and those of *G. parasuis* the lowest, but for individual antibiotics there were several exceptions. For *B. bronchiseptica* interpretive criteria are available for four antimicrobial agents allowing some data interpretation. Susceptibility results are extremely variable (Supplementary Table S10). All *B. bronchiseptica* isolates were resistant to amoxicillin, and 51.1% were resistant to florfenicol whereas 48.9% were florfenicol-intermediate susceptible. For both compounds none of the isolates was susceptible. In contrast, the isolates were merely susceptible to the macrolide antibiotics tildipirosin and tulathromycin. The lack of defined CLSI breakpoints for *G. parasuis* hampered a comparison of the percentages of resistance to the other porcine pathogens.

### Multidrug-resistance

Of the collection of 929 isolates in total, 17 isolates (1.8%) were MDR (Table 1). For *P. multocida* (cattle) and *M. haemolytica,* nine (6.2%) and three (2.3%) MDR isolates were recorded, respectively, and for pigs one *A. pleuropneumoniae* (0.7%) and four (1.7%) *S. suis* isolates were MDR. None of the isolates of the other bacterial species were MDR.

**Table 2:** Prevalence of antibiotic resistance (%) in bacteria isolated from the respiratory tract 2009–2012, 2015–2016 and 2019-2020^a^.

Antimicrobial agent	Cattle									Pigs								
	*P. multocida*			*M. haemolytica*			*H. somni*			*P. multocida*			*A. pleuropneumoniae*			*S. suis*		
	2009–12 (*n* = 134)	2015–16 (*n* = 155)	2019–20 (*n* = 145)	2009–12 (*n* = 149)	2015–16 (*n* = 91)	2019–20 (*n* = 133)	2009–12 (*n* = 66)	2015–16 (*n* = 35)	2019–20 (*n* = 29)	2009–12 (*n* = 152)	2015–16 (*n* = 170)	2019–20 (*n* = 149)	2009–12 (*n* = 158)	2015–16 (*n* = 164)	2019–20 (*n* = 151)	2009–12 (*n* = 151)	2015–16 (*n* = 131)	2019–20 (*n* = 232)
Ceftiofur	0.0	0.0	0.0	0.0	0.0	0.0	0.0	0.0	0.0	0.0	0.0	0.0	0.0	0.0	0.0	2.0	3.1	1.7
Enrofloxacin	3.0	0.7	1.4	0.7	2.2	0.8	0.0	0.0	0.0	0.0	0.6	1.3	1.3	1.2	0.0	0.7	3.8	1.7
Florfenicol	0.0	3.2	2.1	0.0	2.2	2.3	0.0	0.0	0.0	0.7	2.3	0.7	0.6	0.0	1.3	0.0	2.3	0.0
Tetracycline	11.2^b^	11.6^c^	29.7^b,c^	12.1	17.6	19.6	3.0	0.0	3.5	20.4^b^	10.5	11.4^b^	23.4^b^	21.3	13.9^b^	88.1	82.4	83.2
Gamithromycin	1.5^b^	1.3^c^	11.0^b,c^	2.7	7.7	2.3	0.0	0.0	0.0	–	–	–	–	–	–	–	–	–
Tilmicosin	–	–	–	4.0	8.8^c^	2.3^c^	–	–	–	2.0	0.0	0.7	0.6^b^	0.0^c^	11.3^b,c^	–	–	–
Tulathromycin	2.2^b^	1.3^c^	8.3^b,c^	2.7	5.5	1.5	0.0	0.0	0.0	0.0	0.0	0.7	0.0	1.2	0.0	–	–	–
Spectinomycin	6.0^b^	6.5^c^	22.1^b,c^	0.0	3.3	2.3	4.5	0.0	3.5	–	–	–	–	–	–	–	–	–

^a^For CLSI resistance breakpoints see Supplementary Tables S4–S9. A dash indicates no resistance percentage available due to the lack of breakpoints. ^b^indicates significantly different values between 2019–20 and 2009–12. ^c^indicates significantly different values between 2019–20 and 2015–16.

### AMR trends over time

In order to determine whether the resistance percentage has changed over time, the present results of 2019–2020 for 5 major pathogens and for the antibiotics having CLSI breakpoints and commonly tested in the three periods were compared to the resistance percentages observed in VetPath 2009–2012 and 2015–2016 (Table 2). For bovine *P. multocida* the resistance percentage has significantly (*p* ≤ 0.05) increased for the macrolides, spectinomycin and tetracycline in the 2019–2020 period. In contrast, for *M. haemolytica* and *H. somni* the resistance levels remained unchanged, except a significant decrease for tilmicosin in case of *M. haemolytica*. In general, for the porcine pathogens the prevalence of resistance has remained unchanged or was decreased significantly in 2019–20 for tetracycline for *P. multocida* and for *A. pleuropneumoniae* (*p* ≤ 0.05). Only for tilmicosin a significant increase was observed in *A. pleuropneumoniae* (*p* ≤ 0.05). For all other antibiotics the level of resistance remained unchanged.

**Table 1:** Prevalence of bacterial isolates with a multidrug-resistance (MDR) pattern presented by animal and bacterial species.

Animal species	Bacterial species	MDR pattern	MDR number of isolates	MDR number per species	Percentage per species
Cattle	*P. multocida*	ffn, spe, tet	2		
		spe, tet, tul	7		
				9	6.2%
	*M. haemolytica*	enr, ffn, pen, tet, tul	1		
		ffn, pen, tet	1		
		ffn, spe, tul	1		
				3	2.3%
	*H. somni*		0	0	0.0%
				
Total				12	3.9%
Pigs	*P. multocida*		0	0	0.0%
	*A. pleuropneumoniae*	ffn, tet, tia	1	1	0.7%
	*B. bronchiseptica*		0	0	0.0%
	*S. suis*	cef, enr, pen, tet	1		
		cef, pen, tet	3		
				4	1.7%
				
Total				5	0.8%

cef, ceftiofur, enr, enrofloxacin, ffn, florfenicol, pen, penicillin G, spe, spectinomycin, tet, tetracycline, tia, tiamulin, tul, tulathromycin.

### Country comparisons

**Figure 1:**
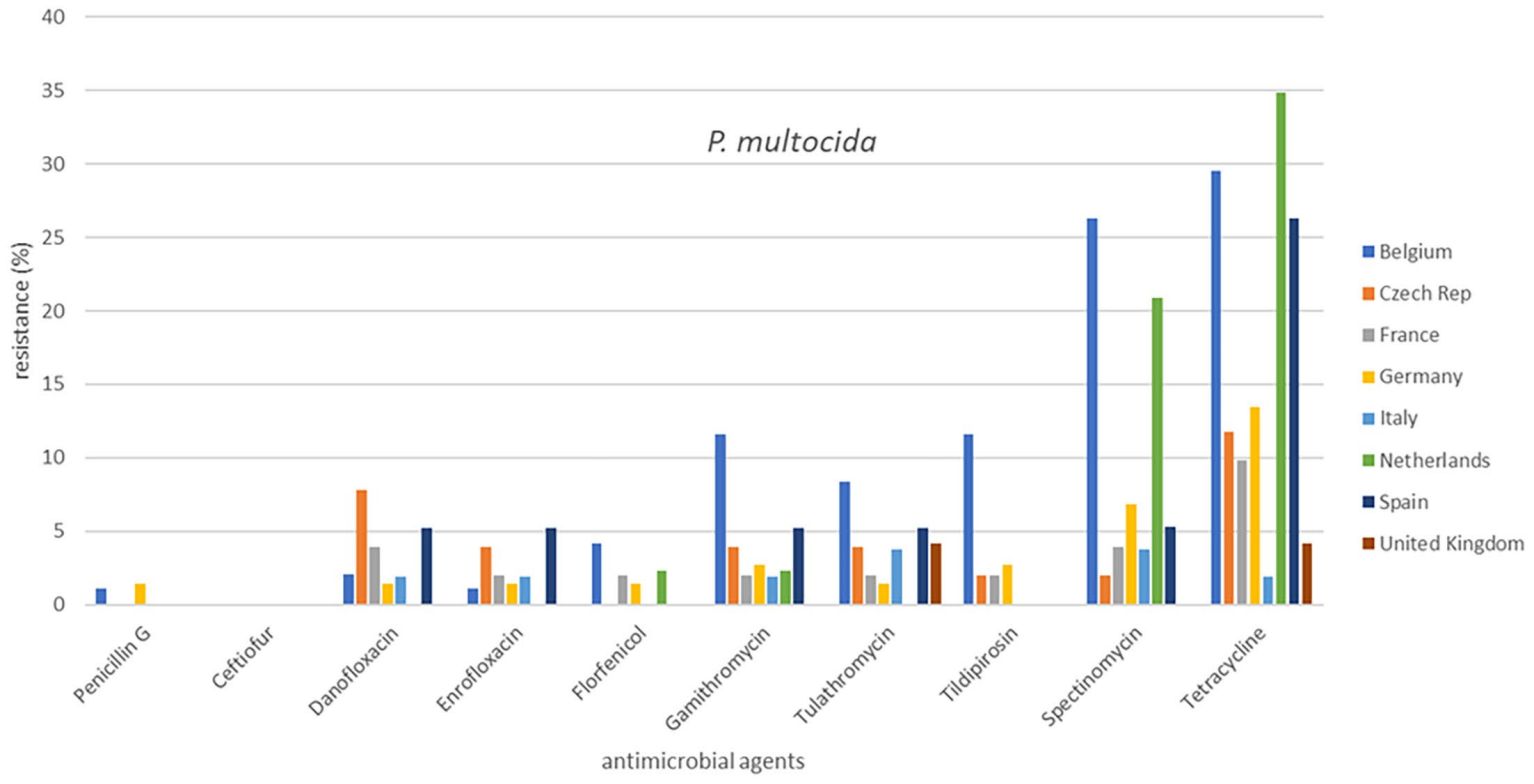
Percentage resistance of cattle pathogens over eight countries from 2009 to 2020.

**Figure 2:**
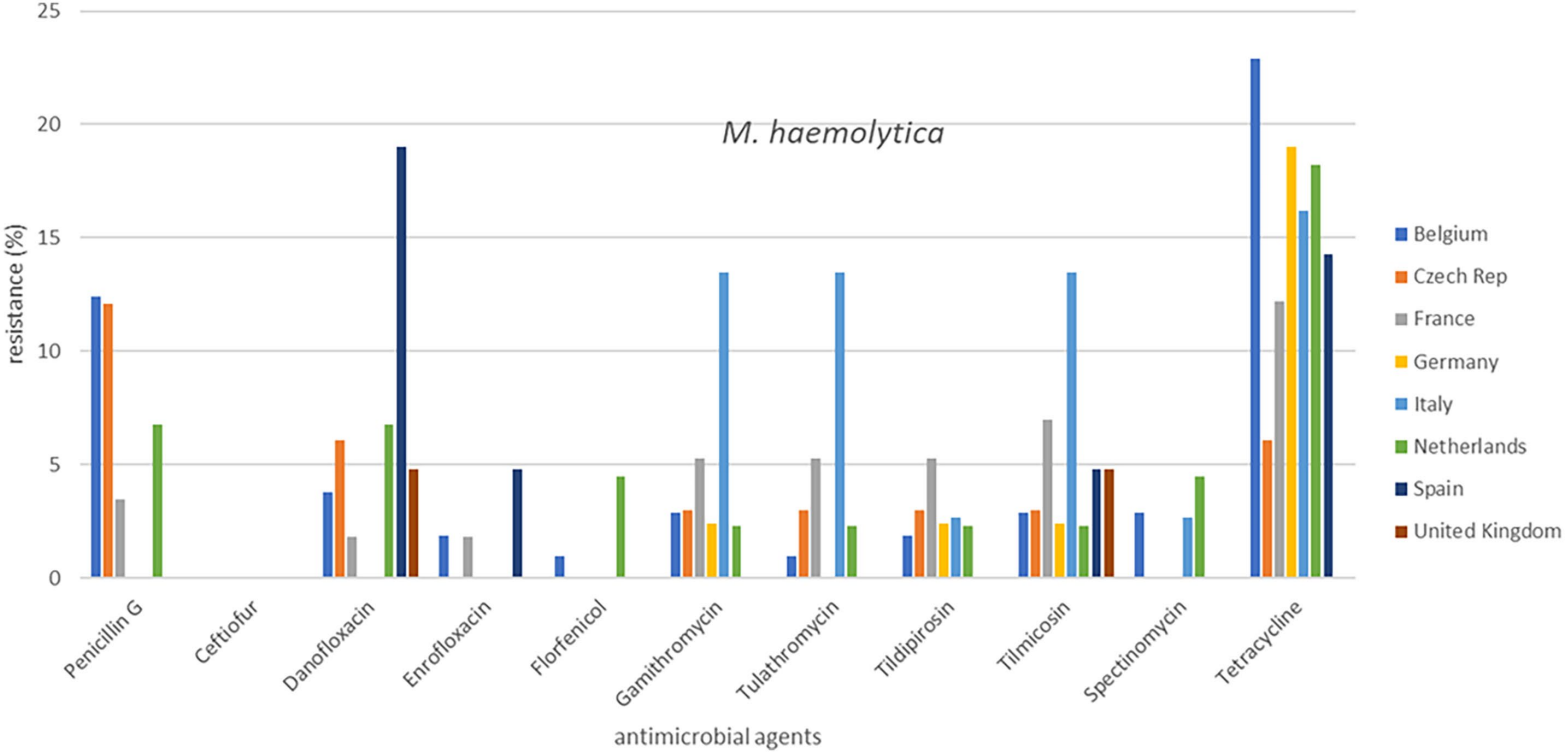
Percentage resistance of cattle pathogens over eight countries from 2009 to 2020.

**Figure 3:**
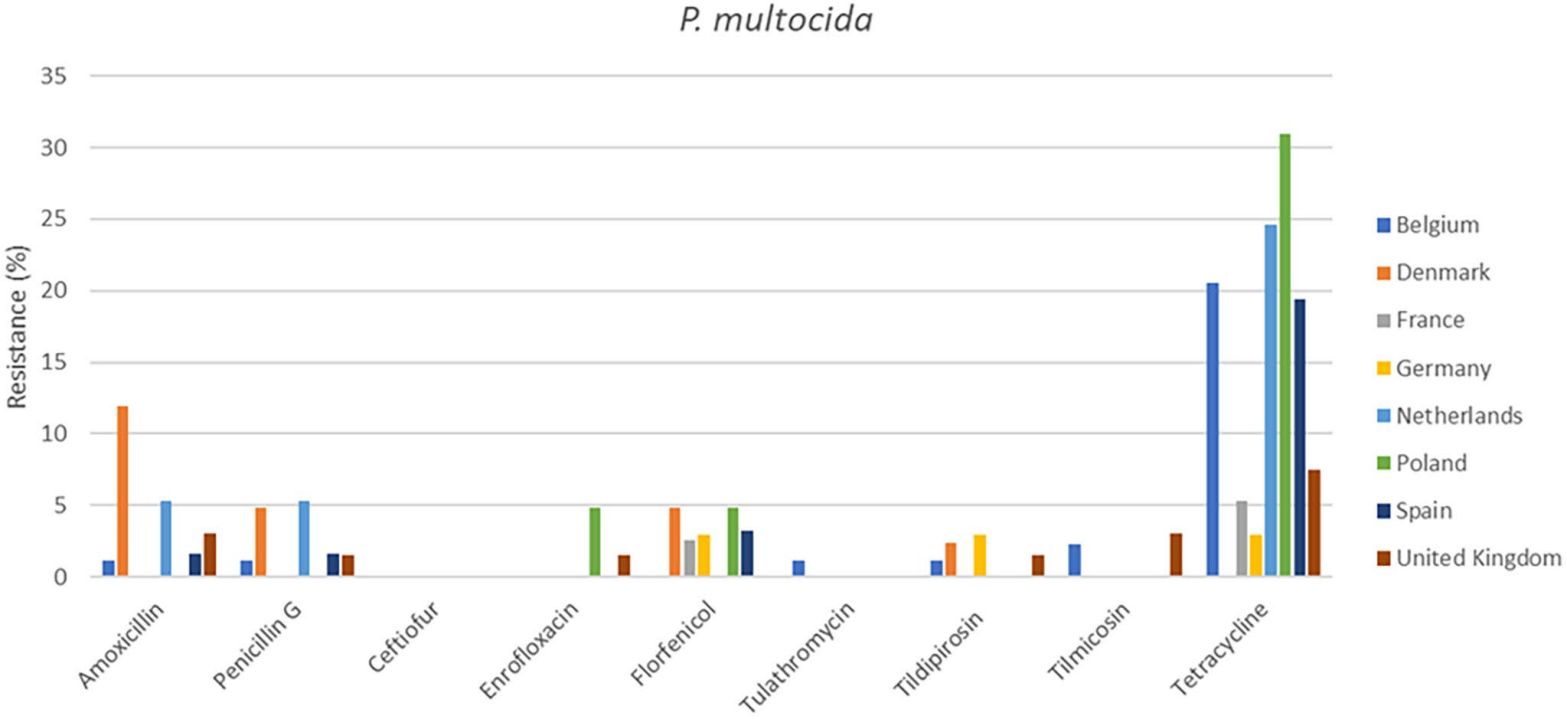
Percentage resistance in pig pathogens over eight countries from 2009 to 2020.

**Figure 4:**
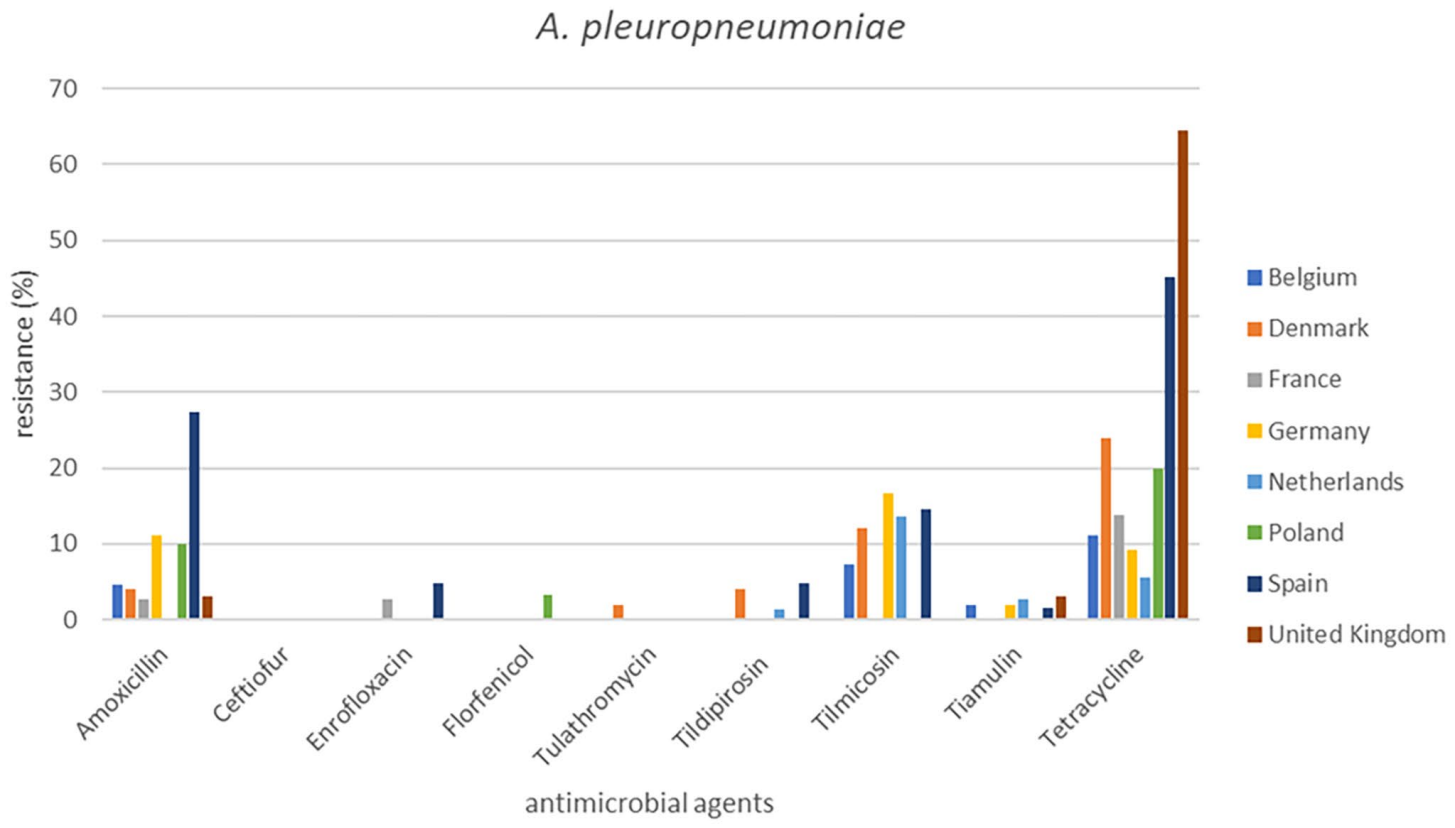
Percentage resistance in pig pathogens over eight countries from 2009 to 2020.

**Figure 5:**
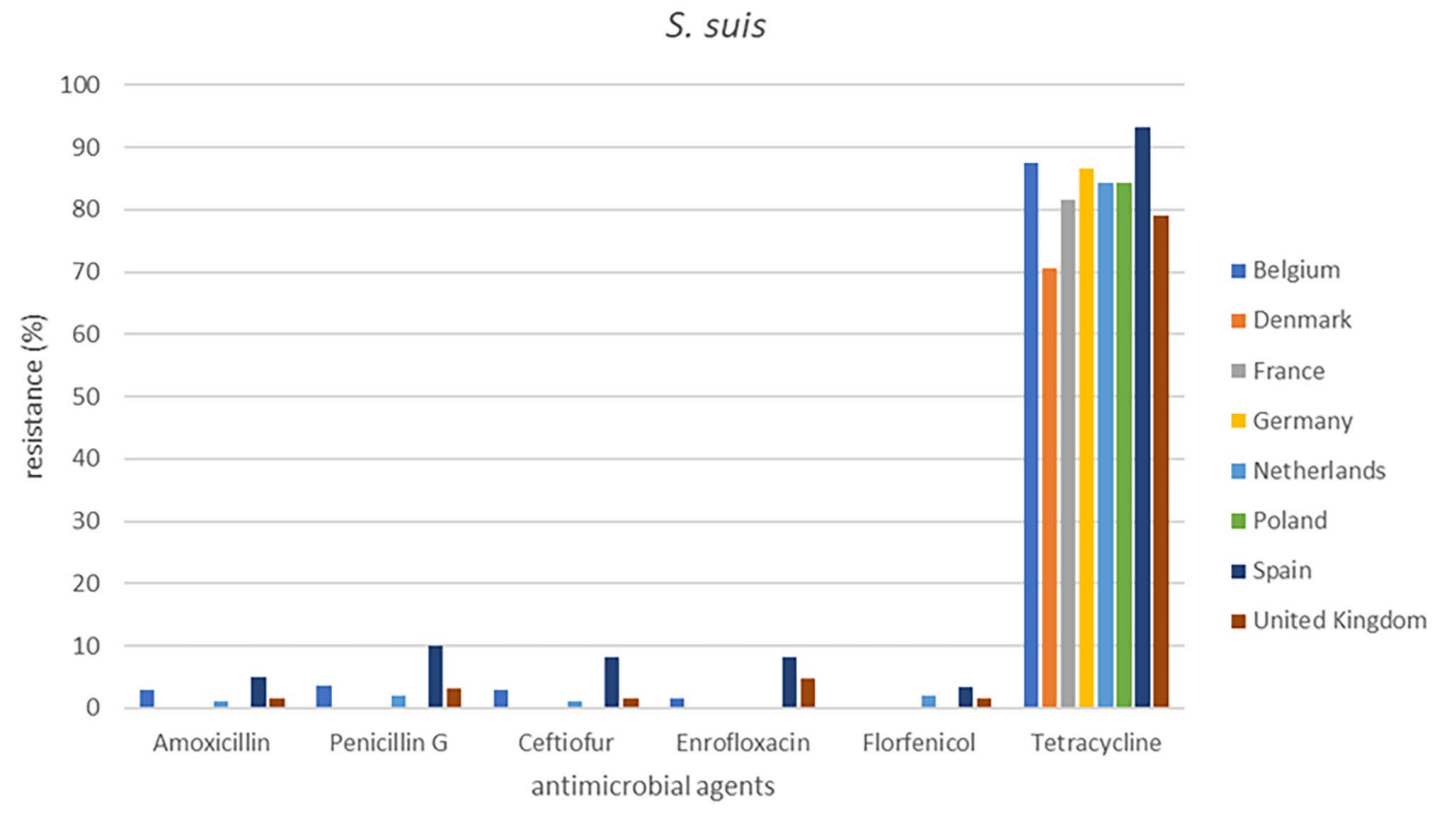
Percentage resistance in pig pathogens over eight countries from 2009 to 2020.

Out of a total of 2,265 isolates, 2,193 were available for analysis of the period 2009 to 2020. The total numbers of isolates per species varied from 360 to 514. The individual isolate numbers per country varied from 20 to 136 in the analysis (Supplementary Table S1). The results are presented in Figures 1, 2 (cattle pathogens) and Figures 3–5 (pig pathogens); individual country resistance percentages are in respective Supplementary Tables S2, S3.

Overall bovine *P. multocida* resistance levels were very low or low, except for spectinomycin (11.0%) and tetracycline (17.3%). Some differences among countries seem apparent (Supplementary Table S2). Spectinomycin and tetracycline resistance percentages in Belgium and the Netherlands differed significantly from several other countries whereas for these two compounds AMR in UK was particularly low (*p* ≤ 0.05). A slightly different picture was observed for *M. haemolytica:* moderate AMR was only observed for tetracycline (16.1%). The percentages resistance of various compounds in UK were the lowest, whereas a few significant differences were observed among the other countries. For instance, the percentage of danofloxacin resistance was significantly higher in Spain; macrolide resistance was significantly higher in Italy and tetracycline resistance was significantly the highest in Belgium (*p* ≤ 0.05; Figures 1, 2).

For porcine *P. multocida* also minor differences of resistance percentages were seen among countries and overall AMR was very low or low (Supplementary Table S3). Exception is tetracycline (14.2%), where various significant differences among countries were observed. Surprisingly, for *A. pleuropneumoniae* tetracycline resistance percentages for Spain (45.2%) and UK (64.5%) were significantly higher than for most other countries. Similarly, significant changes for Spain were observed for amoxicillin, enrofloxacin, and tilmicosin but tetracycline AMR of Denmark (24.0%) was also significantly higher than from the Netherlands. For six antimicrobials AMR could be determined for *S. suis.* For *S. suis* resistance to tetracycline was extremely high in all eight countries, varying from 70.5 to 93.3%. Spain exhibited the highest level of tetracycline resistance (93.3%), and Denmark displayed the lowest tetracycline resistance (*p* ≤ 0.05) (Supplementary Table S3). Resistance percentages of ceftiofur and enrofloxacin in Spain also differed significantly from those in Belgium and the Netherlands (Figures 3–5).

## Discussion

In this study, we aimed to assess the AMR patterns of 307 BRD-associated and 659 SRD-associated bacteria from European cattle and pigs with pneumonia. We also studied the evolution in time of AMR and a cross-country AMR analysis was included. For the great majority of the pathogens it appears that AMR to all relevant antimicrobials (exception is tetracycline) is not expected to be a concern. For the first time, this survey comprises a systematic analysis of cross-country resistance differences. Significant AMR differences were demonstrated to exist among countries for some antibiotics including tetracycline, which shows the potential to contain AMR in part of the European countries.

Knowledge of the resistance profiles of pathogens present in the population is important for making informed decisions about which antibiotic to choose in field infection situations as first-line treatment. Antimicrobial susceptibility testing prior to the treatment of clinical infections is of major relevance in veterinary medicine as it has profound implications for treatment options. Hence, *in vitro* determination of the antimicrobial susceptibility of the pathogenic isolates is of utmost importance and ideally should be conducted prior to treatment. However, in the case of acute infections it is important to use an effective antimicrobial treatment as early as possible. If needed, therapy can be changed later once the susceptibility results are available. Empirical treatment is generally based on knowledge of susceptibility patterns of the different bacterial pathogens to antimicrobial agents used in the animal species. Hence, detailed data on the susceptibility of major pathogens is a pre-requisite, which applies to several local regions and countries due to differences in epidemiology and animal density. In the context of the responsible and sustainable use of antibiotics, resistance monitoring has repeatedly been emphasized as a key tool to detect and follow the emergence of AMR in addition to providing veterinarians data to optimize antibiotic therapy. However, there is a shortage of information regarding the resistance prevalence among disease-causing bacteria from food-producing animals, and the little information available has not necessarily been generated using harmonized methods, thereby limiting data comparability. The long-standing VetPath project, over two decades, described herein, is the only international antimicrobial susceptibility monitoring program for food-producing animals using standardized methods and centralized MIC determination (de Jong et al., 2013; de Jong et al., 2025).

For the current work, CLSI veterinary breakpoints have been defined for about half of the compounds tested, however, in the absence of CLSI clinical breakpoints it was not possible to categorize isolates as susceptible or resistant. Consequently, the MIC data for the antibiotics without internationally recognized breakpoints were difficult to interpret. It is, therefore, important to present the MIC frequency distributions (Supplementary Tables S4–S11) to allow retrospective data interpretation (Schwarz et al., 2010). Providing MIC frequencies also allows to describe the MIC patterns, e.g., monomodal or bimodal distributions. Unless otherwise stated, the susceptibility data of studies quoted below refer only to MIC values and have only been interpreted by CLSI-defined veterinary breakpoints.

In cattle, the majority of BRD cases are associated with *P. multocida* and *M. haemolytica* infections, in addition, we were also able to isolate the less prevalent pathogen found in Europe *H. somni* (Ånestad et al., 2025). The present study demonstrates that, taken as a whole, antimicrobial susceptibility of these organisms in Europe is high for almost all antibiotics licensed for BRD. Since the MIC distributions of cefquinome and marbofloxacin are similar to ceftiofur and enrofloxacin, respectively, the results of this study indicate that for these drugs clinical resistance of all three pathogens is infrequently observed. Likewise, almost all isolates tested were susceptible to florfenicol. Recently the VetCAST Committee has published clinical breakpoints for florfenicol for bovine respiratory pathogens (*P. multocida* >1 μg mL^−1^; *M. haemolytica* >2 μg mL^−1^) (Veterinary European Committee on Antimicrobial Susceptibility Testing, 2019). Application of these new breakpoints did not change the resistance percentages based on the CLSI breakpoints (*P. multocida* >4 μg mL^−1^; *M. haemolytica* >4 μg mL^−1^). Resistance to the newer macrolide antibiotics gamithromycin, tildipirosin and tulathromycin, varied from 1.5 to 11.0%. In contrast, for older compounds, such as tetracycline, resistance was observed and varied depending on bacterial species. It is noted that for some antibiotics without defined CLSI breakpoints, MIC distribution was bimodal suggesting presence of resistance or of non-wild type isolates.

Similar results have been reported for *P. multocida* in the GE*RM*-Vet program covering the time period 2016–2020 (GE*RM*-Vet, 2022). In the GE*RM*-Vet program (reporting on annually 75–192 *P. multocida* respiratory isolates), resistance to ceftiofur, enrofloxacin, florfenicol, penicillin and tulathromycin in *P. multocida* was comparable to our results. MDR amounted to 5.6% in the GE*RM*-Vet program. In Sweden, the SVARM program reported full susceptibility (based on both CLSI-clinical breakpoints and ECOFFs) to ceftiofur, enrofloxacin, florfenicol and tetracycline among *P. multocida* isolates recovered from calves in 2022–2023 (SVARM, 2024). In contrast, resistance to penicillin, the first-choice antibiotic for pneumonia in cattle in Sweden, amounted to 10.3%, and resistance to gamithromycin was 1.5%. In a Norwegian monitoring study on calves (Ånestad et al., 2025), all *P. multocida* isolates were susceptible to several antibiotics including penicillin, enrofloxacin, florfenicol and tetracycline. Similarly, negligible levels of resistance were observed in the Finnish national monitoring project, except for (oxy) tetracycline with a proportion from 1 to 8% (FINRES-Vet, 2024). In the UK surveillance program, resistance of *P. multocida* isolates was for most compounds tested not detected or much lower, but for spectinomycin and tetracycline very high resistance rates were observed exceeding 30 and 50%, respectively (UK-VARSS, 2024). In addition to the aforementioned longitudinal national surveillance projects, several *ad hoc* single studies are available. A German study on *P. multocida* isolates recovered in Bavaria (n = 345) reported low percentages of resistance for ceftiofur, enrofloxacin, florfenicol, and penicillin (0.0–2.9%), but resistance to tetracycline (38.0%), and spectinomycin (32.8%) was high, while tulathromycin resistance amounted to 13.0% and MDR (based on non-susceptibility) amounted to 13.9% (Melchner et al., 2021). These results are comparable with those of older German studies (Wallmann et al., 2004). In contrast to the above observations, *P. multocida* isolates recovered from Spanish feedlot cattle displayed markedly higher AMR for various compounds (Serna et al., 2025) including fluoroquinolones (4.0–14.7%), macrolides (9.3–28.0%) and tetracycline (32.0%), but no resistance to ceftiofur and penicillin was observed. The relatively low resistance levels to various antimicrobials contrast with the more common occurrence of resistance in a few European countries (Lachowicz-Wolak et al., 2025; Schönecker et al., 2020). In Poland marked resistance to macrolides (22.9–25.0%), beta-lactams including ceftiofur (14.6–18.8%), spectinomycin (25.0%) and tetracyclines (79.2–81.3%) was recorded in dairy calves; resistance exclusively to florfenicol and enrofloxacin was low (<2.1%) (Lachowicz-Wolak et al., 2025). It should be noted that Poland has one of the highest levels of antimicrobial use in food-producing animals among EU countries (European Medicines Agency, 2023). In Switzerland in a study on *P. multocida* isolates from 12 veal calf farms, extremely high percentages of resistance were recorded for oxytetracycline and spectinomycin, and resistance to most other antibiotics was also much higher than in our study. Exceptions were enrofloxacin and florfenicol (Schönecker et al., 2020).

Similar observations, or even lower resistance percentages than in our study, were made for *M. haemolytica* (GE*RM*-Vet, 2022), but the GE*RM*-Vet program recorded in 2020 (*n* = 100) higher levels of resistance to penicillin (31.0%) and lower levels of tetracycline resistance (11.0%). Moreover, for enrofloxacin, penicillin and tilmicosin moderate levels of AMR were noted. SVARM reported for *M. haemolytica* isolates full susceptibility to enrofloxacin, florfenicol and tetracycline, but 12.5% resistance to penicillin (SVARM, 2024). Similarly, in Norway *M. haemolytica* isolates were susceptible to all commonly used antimicrobials for BRD treatment, except 4.1% resistance to penicillin from two calves of the same herd was recorded (Ånestad et al., 2025). FINRES-Vet (2024) reported an absence of resistance to ampicillin, enrofloxacin, florfenicol and tulathromycin, but resistance to oxytetracycline and penicillin amounted to 4.9 and 9.8% in 2023, respectively. In the UK-VARSS program resistance of the antibiotics tested was low (<4.2%) or not detected (UK-VARSS, 2024). Our results are also in agreement with older German studies (Schwarz et al., 2004), who reported for instance an absence of resistance to ceftiofur and florfenicol and a low rate of resistance to spectinomycin (1.4%) for a collection of *M. haemolytica* obtained from various locations of Germany. The more recent German study of Melchner et al. (2021) on *M. haemolytica* isolates (*n* = 273) reported low percentages of resistance for ceftiofur, enrofloxacin, florfenicol, penicillin and tilmicosin (0.0–3.7%) but resistance to spectinomycin (10.3) was markedly higher than in our study. Moreover, the percentage intermediate to spectinomycin was extremely high (70.7%) (Melchner et al., 2021). Tetracycline resistance amounted to 21.3% in accordance with our results. MDR was 5.1%. In a study on the genetic basis of macrolide resistance in *M. haemolytica* isolates it was concluded that macrolide resistance rates in Germany are very low (Kostova et al., 2024). In contrast, in Poland very high rates of resistance were observed for ceftiofur (31.8%), penicillins (63.6%), macrolides (63.6%), and tetracyclines (45.5%), whereas resistance rates to enrofloxacin, florfenicol and spectinomycin were only slightly higher compared to those in our study (Lachowicz-Wolak et al., 2025). Note, however, that the percentage of intermediate susceptibility for the latter two compounds amounted to 50%. In Switzerland, elevated percentages of resistance were observed for penicillin (26%) and oxytetracycline (27%) (Schönecker et al., 2020). Different sampling strategies such as more than one sample per farm, sampling of cattle with unknown antimicrobial treatment history and deviating local antimicrobial consumption of the farms included, may explain the differences compared to our program.

In the current study the pathogen with the lowest percentage of resistance was *H. somni*, with very low MIC values. European data on the antimicrobial susceptibility of *H. somni* are rare and almost none of the aforementioned studies included *H. somni* (Ånestad et al., 2025). Nevertheless, *H. somni* has been confirmed to be a very susceptible organism, as described by some studies. Aarestrup et al. (2004) reported that all *H. somni* isolates (n = 80), from diagnostic submissions of 80 different Danish cattle herds, were fully susceptible to all antimicrobials tested, including ceftiofur, ciprofloxacin, florfenicol, penicillin, spectinomycin and tetracycline. In another Danish study, *H. somni* isolates recovered from broncheoalveolar lavage fluid of veal calves were all susceptible to the most commonly used antimicrobials in Denmark such as ampicillin, penicillin, ceftiofur, florfenicol, spectinomycin, tilmicosin or tulathromycin (Kudirkiene et al., 2021). In *H. somni* isolates from veal calves only resistance to ampicillin (42%) and penicillin (23%) was recorded (Schönecker et al., 2020). FINRES-Vet (2024) reported out of six antimicrobials tested, only resistance to oxytetracycline was noted at low levels. In the UK-VARSS program *H. somni* isolates were recovered infrequently, e.g., three in 2022 and 27 in 2023; all isolates were fully susceptible to all antimicrobials tested (UK-VARSS, 2024). Similar findings were described for non-European collections (Goldspink et al., 2015; Holschbach et al., 2020; Ueno et al., 2022). In an Australian study (Goldspink et al., 2015), an absence of resistance to ceftiofur, enrofloxacin, florfenicol, tilmicosin and tulathromycin was observed, whereas tetracycline resistance was limited to only one out of 53 isolates (1.9%). In a Japanese study on 166 *H. somni* isolates, the same outcome was reported, but tulathromycin was not tested (Ueno et al., 2022). In a Mid-West American study, *H. somni* isolates (*n* = 749) exhibited the highest susceptibility among several respiratory pathogens recovered in dairy cattle (Holschbach et al., 2020). Among 18 antimicrobials tested the lowest percentages of resistance were observed for ceftiofur (2%), florfenicol (3%), enrofloxacin (5%), tildipirosin (5%) and gamithromycin (6%).

Regarding pigs, five major respiratory tract pathogens, *P. multocida, A. pleuropneumoniae, S. suis, G. parasuis,* and *B. bronchiseptica*, were investigated. The results suggest a high level of susceptibility to most of the compounds tested. For *P. multocida*, resistance varied from 0.0 to 3.5%, with the exception of tetracycline (10.6%). GE*RM*-Vet has reported similar results for most compounds (*n* = 442; <1% resistance), but resistance to tetracycline amounted to 17.0% (Wallmann et al., 2004). Another GE*RM*-Vet study described *in vitro* susceptibility to 24 antimicrobial agents in 1,111 *P. multocida* isolates collected from pigs in Germany between 2004 and 2006 (Kaspar et al., 2007). Using current breakpoints, resistance to ceftiofur, enrofloxacin, florfenicol, and tulathromycin was zero or below 1% but resistance to tetracycline varied from 11.5% in breeding pigs to 19.2% in piglets. Similar results were reported by the national survey of France (Resapath; based on disk diffusion methodology) and in the national collection of UK (Anses, 2024; UK-VARSS, 2024). In the national survey of Sweden, the antimicrobial susceptibility of *P. multocida* has also been followed for many years. Generally, the occurrence of AMR has been rare in the past decade (SVARM, 2024). For instance, for *P. multocida* isolates resistance was absent to all relevant antibiotics during 2014–2023 (SVARM, 2024). Heuvelink et al. (2015) reported full susceptibility to enrofloxacin and florfenicol for a Dutch collection of 272 *P. multocida* of 2013–2014, whereas tetracycline and tilmicosin resistance was 12.9 and 44.5%, respectively. In contrast, Nedbalcová and Kučerová (2013) reported in the Czech Republic slightly higher resistance (0.6–4.5%) to above antibiotics tested in GE*RM*-Vet, and for tetracycline as well (32.2%). In Romania, in a monitoring study based on disk diffusion methodology, resistance proportions of *P. multocida* isolates (n = 121) were slightly higher than in our study, but for tilmicosin 28.9% resistance was recorded (Siteavu et al., 2023). Another study described the susceptibility to various antibiotics in 132 *P. multocida* isolates collected in Spain (Vera Lizarazo et al., 2006). The authors recommended ceftiofur, enrofloxacin or florfenicol for treatment of infections caused by *P. multocida* due to the absence of *in vitro* resistance. Similar results were noted in another Spanish study on *P. multocida* isolates from 2017–2019 (*n* = 130) (Vilaró et al., 2020); resistance to ceftiofur, enrofloxacin, florfenicol, tildipirosin and tulathromycin was 0.0 to 2.3%. In a more recent study based on non-susceptibility percentages of *P. multocida* isolates from 2019–2022 (*n* = 536), these authors confirmed the preceding results (Vilaró et al., 2023). Recent results from Australian studies on *P. multocida* isolates of diagnostic specimens are in line with our results; high percentages of resistance (23.3–27.5%) were only recorded for tetracycline in both studies (Dayao et al., 2014a; Truswell et al., 2023).

For *A. pleuropneumoniae* isolates, resistance to ceftiofur, enrofloxacin, florfenicol, tiamulin and tulathromycin in our study was 0.0–2.0%, which is comparable to the findings on German isolates (0.0–2.3%; n = 196) over the years 2016–2020 (GE*RM*-Vet, 2022). In both surveys moderate levels of tetracycline resistance were observed for *A. pleuropneumoniae*, and in the GE*RM*-Vet study the percentage intermediate to tetracycline was very high. In the national survey of Sweden, the only resistance of *A. pleuropneumoniae* isolates (*n* = 42) was observed to florfenicol (2.4%) during 2022–2023 (SVARM, 2024). FINRES-Vet (2024) reported an absence of resistance to ceftiofur, florfenicol, tiamulin and tulathromycin; resistance to oxytetracycline and penicillin was 3.8% in 2023. Similar results were observed in the national surveys of Denmark and France (DANMAP, 2024; Anses, 2024), except higher rates of resistance to tetracycline in Resapath. Several *ad hoc* single studies are also available on the susceptibility to *A. pleuropneumoniae*. Hennig-Pauka et al. (2022) reported a very low percentage of AMR in a collection of 1,647 unique isolates from North Western Germany (ceftiofur, 0.1%; enrofloxacin, 0.8%; florfenicol, 0.1%; tiamulin, 2.3%; tilmicosin, 2.4%; tulathromycin, 0.0%), with the exception of penicillin (9.0%) and tetracycline (14.8%). Moreover, the percentage of intermediate isolates was very high for the two latter compounds (42.8 and 63.6%, respectively). Interestingly, the MIC values for ceftiofur were clearly higher than our MICs (albeit almost all isolates were still susceptible), whereas all MICs of the other compounds were comparable to the MICs in our dataset. In Romania, resistance percentages of *A. pleuropneumoniae* isolates (*n* = 137) were (based on disk diffusion) similar or slightly higher, except for tilmicosin (38.0%) (Siteavu et al., 2023). Kucerova et al. (2011) reported very low percentages of AMR in 242 isolates from the Czech Republic (ceftiofur, 0.0%; enrofloxacin, 0.8%; florfenicol, 0.8%; tilmicosin, 1.2%; tiamulin, 1.7%; tulathromycin, 0.0%), with the exception of tetracycline (24.0%). Similar results were reported in Switzerland, but with 8.4% resistance to tetracycline (Matter et al., 2007). In the latter study, using currently adopted CLSI breakpoints, an absence of resistance to ceftiofur and florfenicol, and a low resistance to enrofloxacin (1.2%) and tilmicosin (2.4%) was observed, while tiamulin resistance amounted to 10.8%. In The Netherlands, resistance of *A. pleuropneumoniae* (*n* = 248) to enrofloxacin and florfenicol was absent or very low, but for tetracycline, tilmicosin and tiamulin resistance rates of 19.0, 14.1 and 40.3%, respectively, were reported (Heuvelink et al., 2015). In a Spanish study on 162 *A. pleuropneumoniae* isolates low percentages of resistance were observed; for ceftiofur 0.0%, florfenicol 3.0%, tiamulin 1.2%, tilmicosin 0.6%, and for both tildipirosin and tulathromycin 0.0%, but for enrofloxacin 11.1% and amoxicillin 24.7% resistance was reported; penicillin and tetracycline were not tested (Vilaró et al., 2020). In a Danish study of *A. pleuropneumoniae* collected in the study period 2004–2017, almost all isolates were susceptible to all compounds tested including ceftiofur, ciprofloxacin, florfenicol, tiamulin, and the macrolides tilmicosin and tulathromycin (Holmer et al., 2019). One exception: tetracycline resistance amounted to 5.8%.

Infections with *S. suis,* another important pig pathogen (Dechêne-Tempier et al., 2021), are traditionally treated with penicillins (first choice antimicrobial). For *S. suis,* CLSI have approved breakpoints for six antimicrobials tested in the current study. In The Netherlands a large collection of *S. suis* isolates (*n* = 1,163) recovered from post-mortem pig samples was analyzed for its antimicrobial susceptibility (Van Hout et al., 2016). Resistance to ampicillin, penicillin, ceftiofur, enrofloxacin and florfenicol was <0.6%, but resistance to tetracycline was 78.4%, which is similar to the percentage resistance in the preceding VetPath study (82.4%; De Jong et al., 2023) and the present study (83.2%; in the Netherlands 84.2%; Supplementary Table S3). In the Czech Republic a collection of 506 field isolates of *S. suis* from the period 2018–2022 were tested for susceptibility to a panel of 12 antimicrobials (Nedbalcova et al., 2022). Resistance to ampicillin, ceftiofur, enrofloxacin and florfenicol was 0.0–0.4%; for penicillin resistance amounted to 5.6% and for tetracycline to 53.4%. In Romania, resistance proportions of *S. suis* isolates (*n* = 207) to ampicillin, penicillin, ceftiofur, enrofloxacin and florfenicol were low based on disk diffusion methodology, but 73.9% resistance to tetracycline was observed (Siteavu et al., 2023). In a collection of 405 *S. suis* isolates from the United Kingdom of both disease-associated and non-disease-associated provenance, resistance proportions were similar for the five above antimicrobials (0.0–0.7%), for penicillin 4.7% and for tetracycline 90.9% (Hernandez-Garcia et al., 2017). Similar data were noted in a Swedish collection of 188 *S. suis* isolates in grower pigs from herds with and without *S. suis* associated disease (Werinder et al., 2020). Low levels of resistance were found for enrofloxacin (2.1%) and penicillin (0.0%), whereas in pigs with a clinical history 8.5 and 7.7%, respectively, were observed. In both groups resistance to tetracycline was very high (85.1 and 91.5%). SVARM reported for Swedish *S. suis* isolates (*n* = 72) 0% resistance to enrofloxacin and 4.2% to penicillin G based on currently adopted CLSI breakpoints over the time period 2019–2023, but for tetracycline 62.5% resistance was recorded (SVARM, 2024). For Germany slightly lower resistance proportions were reported in the BfT-Germ*-*Vet project (0.0% for all five compounds) (Schwarz et al., 2007). In the GE*RM*-Vet project (GE*RM*-Vet, 2022), resistance to ampicillin, ceftiofur and enrofloxacin was 1.2–2.1%, resistance to penicillin 8.3% and tetracycline resistance 77.6% (*n* = 483), all in accordance with our findings for Germany (Supplementary Table S3). Other European surveys have confirmed the extremely high levels of resistance to tetracycline (e.g., Anses, 2024; UK-VARSS, 2024). The above results together with our findings (0.0 to 3.0% resistance to five compounds) are compatible with the older results of *S. suis* isolates from seven EU countries (Wisselink et al., 2006).

For *B. bronchiseptica*, CLSI breakpoints are only available for amoxicillin, florfenicol, tildipirosin and tulathromycin. The classification of *B. bronchiseptica* isolates as susceptible, intermediate, or resistant was only possible for amoxicillin, florfenicol and tulathromycin. In our work MIC_50/90_ for beta-lactam agents, lincomycin, spectinomycin, tiamulin and first-generation macrolides were very high. In contrast, fluoroquinolones, colistin, second-generation macrolides and tetracyclines exhibited lower MIC_50/90_ values (Supplementary Table S10). Similar results are observed in the GE*RM*-Vet project; low MIC_90_ values were noted for enrofloxacin (0.5 μg mL^−1^), which have been unaltered for the past 8 years (GE*RM*-Vet, 2022). For the beta-lactam agents high MIC_90_ values (>64 μg mL^−1^) were observed as well as 100% resistance to ampicillin, which matches previous reports (Kadlec et al., 2004). In several studies wide variations of florfenicol-resistant or florfenicol-intermediate proportions were noted (Kadlec and Schwarz, 2018). The figures for intermediate and resistant to florfenicol were 17.5 and 2.9% (Kadlec et al., 2004), whereas in our study the percentages were 48.9 and 51.1%, respectively. However, with regard to the lack of susceptibility, overall, these results are in line with the most recent data of the German survey (GE*RM*-Vet, 2022) where the figures for florfenicol-intermediate and resistant isolates were 87.1 and 3.2%, respectively. Vilaró et al. (2020) reported the susceptibility to florfenicol was close to 50%. In comparison, Heuvelink et al. (2015) assessed corresponding figures of 55.9% intermediate and 4.5% resistance to florfenicol. In a study of a collection of 107 German *B. bronchiseptica* pig isolates (Prüller et al., 2015), 0.9% florfenicol resistance and 13.1% intermediate resistant were reported. Moderate rates of florfenicol resistance were also found for 602 isolates from North America (Sweeney et al., 2022). However, the percentage of isolates in the florfenicol-intermediate category amounted to 77.9%. In our study a high susceptibility to the second-generation macrolides tildipirosin and tulathromycin was observed, which is in accordance with other studies (Vilaró et al., 2020; GE*RM*-Vet, 2022; Sweeney et al., 2022).

*Glaesserella parasuis* is an early colonizer of the upper respiratory tract of healthy pigs. Virulent strains of this pathogen can cause Glässer’s disease, characterized by polyarthritis, fibrinous polyserositis, and meningitis, resulting in economic losses and reduced pig welfare (Costa-Hurtado et al., 2020). In the acute form it might be associated with pneumonia and septicemia without polyserositis. Only limited knowledge is available on the antimicrobial susceptibility of *G. parasuis* isolates. Calculation of resistance is precluded because clinical breakpoints have not been defined. Our study confirms the MIC values of other work. The obtained MIC_50_ values in this current study were slightly lower for several antibiotics than those in a study on 2,046 *G. parasuis* isolates recovered in Germany from 2006 to 2021 (Wiencek et al., 2022). In the study of Wiencek et al. and in various other studies breakpoints defined for other organisms have been provisionally applied to interpret the MIC data for *G. parasuis*. In another German study on 123 *G. parasuis* field isolates (Brogden et al., 2018), MIC_50_ values showed generally good accordance with those of the present study, but MIC_90_ values for several compounds were markedly higher in the German study. De la Martín Fuente et al. (2007) have compared the antimicrobial susceptibilities of *G. parasuis* from pigs in Spain and the United Kingdom, based on CLSI breakpoints for *A. pleuropneumoniae*. All the United Kingdom isolates were susceptible to penicillin, ceftiofur, enrofloxacin, florfenicol and tilmicosin, and most of them were susceptible to the remaining antimicrobials. In contrast, all Spanish isolates were only exclusively susceptible to florfenicol, and high proportions of resistance were encountered for the other antimicrobials. A Danish study investigated 52 *G. parasuis* isolates collected in the period 1998–2002, again applying the breakpoints for *A. pleuropneumoniae*, reported similar results: None of the isolates were resistant to 10 antibiotics including ceftiofur, florfenicol, penicillin and tilmicosin (Aarestrup et al., 2004). Nedbalcová and Kučerová (2013) confirmed that based on species non-specific breakpoints resistance in *G. parasuis* was in general lower compared to *P. multocida*. Resistance to ceftiofur, enrofloxacin and florfenicol was absent. In a Chinese study the susceptibility of 117 clinical *G. parasuis* isolates to several macrolide antibiotics was confirmed: MIC_50_ and MIC_90_ values were similar to our MIC_50/90_ values (Zhang et al., 2025). As regards Australian *G. parasuis* isolates (*n* = 97), MIC_50_ values were also in accordance with our results (Dayao et al., 2014b). Application of interpretation criteria developed for other host or bacterial species, as frequently applied in several of above studies (e.g., Aarestrup et al., 2004; De la Martín Fuente et al., 2007; Nedbalcová and Kučerová, 2013; Wiencek et al., 2022), may suggest an AMR issue that actually does not exist. While it is not uncommon to test and report a combination of antimicrobials with both species-specific and non-species-specific (e.g., human) interpretive criteria, these classifications ignore the potential impact of interspecies pharmacokinetics on clinical outcome. In line with Schwarz et al. (2010), Brogden et al. (2018) and Sweeney et al. (2018) we, therefore, do not support the application of species non-specific breakpoints and endorse that the setting of veterinary-specific breakpoints remains of high importance.

In the last decade an increased prevalence of MDR bacteria has been reported. We determined the percentage of MDR for our collection of respiratory isolates, although for only a limited number of antimicrobials clinical breakpoints have been set. MDR was 0–6.2%, on average 1.8%. MDR was significantly lower (*p* ≤ 0.05) for *P. multocida* in the preceding VetPath study of 2015–16 (1.3%); MDR for the other organisms were unaltered. However, it is also apparent that several compounds without breakpoints (e.g., trimethoprim/sulfamethoxazole), exhibited bimodal or multimodal distributions of isolates over the MIC range observed, which suggest resistance mechanisms are present. If breakpoints for antimicrobials of these partly less commonly used or non-licensed classes were available, MDR levels might be higher for some pathogens. This does not apply to other commonly used compounds without breakpoints, because for compounds of these classes usually one representative compound with defined breakpoints is available to define MDR due to cross-resistance (e.g., fluoroquinolones or macrolides). Similar to our analysis, Kucerova et al. (2011) determined resistance phenotype patterns for *A. pleuropneumoniae*, which resulted in an absence of MDR.

Some European studies on the antimicrobial susceptibility of BRD and SRD pathogens have shown that resistance to antimicrobial agents is increasing (Hernandez-Garcia et al., 2017; Holmer et al., 2019; Melchner et al., 2021). In other studies, AMR levels of various antimicrobial agents were rather constant (Van Hout et al., 2016; Nedbalcova et al., 2022; SVARM, 2024). The large German and French government national surveys also suggest that the susceptibility of target pathogens of respiratory tract infections has not changed over the past decade (GE*RM*-Vet, 2022; Anses, 2024), although year-to-year fluctuations are apparent. In other surveys a reduction of AMR has been observed for various antimicrobials (Hennig-Pauka et al., 2022; Holschbach et al., 2020; Wiencek et al., 2022). The present study demonstrates that for most antimicrobial compounds AMR rates have remained stable when compared to the reference periods 2009–2012 and 2015–2016 (Table 2). In a few cases a significant increase or decrease of AMR has been observed in our study.

Our study is also one of the first attempts to perform a cross-country analysis of resistance in clinical isolates across Europe. This work showed substantial variations in percentages of resistance, both between countries and also between bacterial species (Figures 1–5). For several antibiotics significant differences of resistance proportions among countries were observed, particularly for tetracycline. These differences among countries deserve further investigation. In this context, it may be relevant to link the resistance proportions with the national antimicrobial use in animals, i.e., to use the consumption data of the ESVAC project (European Medicines Agency, 2023). However, animal species-specific consumption data for BRD and SRD are not included in the ESVAC reports. It is also of interest to compare our results with the EARS-Vet project which aims to study cross-country resistance differences in Europe (Lagrange et al., 2023). A comparison, however, is currently hampered due to a variety of different variables applied in the EARS-Vet pilot project. According to the authors the project is characterized by highly diverse and fragmented sampling procedures (poor information on animal/herd identifier, production and specimen type), is based on passive data collection, often with limited number of isolates, inclusion of samples from one or more antimicrobial courses prior to sampling, use of highly diverse laboratory techniques (mix of microdilution and disk diffusion technique), and use of differing interpretation criteria (ECOFFs, clinical breakpoints of CLSI, of EUCAST or national breakpoints) (Lagrange et al., 2023). In contrast, our isolates are collected based on identical sampling procedures, susceptibility assessed by broth microdilution MIC determination in one single central laboratory, and application of identical interpretive criteria. Still, the pilot study of EARS-Vet shows what could be achieved in future, and will form an important basis for systematic data collection and analysis.

As applies for any monitoring program, there are some important limitations to the study design. For instance, isolates were from diagnostic samples, and in part from animals with unknown clinical history, which could be a potential bias. Isolates from recently pretreated animals have been consequently excluded from the collection. If samples should have been collected in cases of antibiotic treatment failure or recurrent cases, a more resistant population might be found than that in the general population. Hence, our study may present a worst-case situation, and if this applies, the real rates of resistance in the field would be even lower than described in the tables of our study. This methodological risk has been investigated by Larsen et al. (2015). This research group concluded that antimicrobial susceptibility data based on routine diagnostic specimens are generally biased in favor of resistance and are not representative for patients without a history of antimicrobial therapy. Similar experiences have been made in other studies. Hence, a slight overestimation of resistance cannot be excluded in our work. An additional limitation of our work is that information on breed, type and size of the holding was not always available. The current study examined a total of 966 clinical isolates (29–232 isolates per bacterial species); in the entire VetPath project isolates have continuously obtained over more than two decades from respiratory samples resulting in 4,772 clinical isolates. While this is a substantial number, it is only a very small sample of the total BRD and SRD pathogen population in the EU. Moreover, the numbers of isolates per country as such slightly differed between the preceding surveys and the present VetPath survey. This could hamper the comparison between the time periods because AMR may vary according to geographical location. Care should also be taken when comparing existing data from different studies and different laboratories, as such a comparison can be hampered by inconsistencies in methodology including sampling strategies, selection of antimicrobial substances in the test panel, and variations in interpretation criteria for clinical breakpoints.

In conclusion, the results of this pan-European survey, with standardized methods, show little or no antibacterial resistance to various licensed antibiotics including beta-lactam antibiotics, fluoroquinolones, phenicols and macrolides among the major respiratory tract pathogens recovered from diseased cattle and pigs. Exceptions are older molecules such as tetracycline. Based on the frequent occurrence of resistance, tetracycline should preferably only be used when susceptibility testing has shown efficacy. MDR, referring to antibiotics of seven classes, was low (1.8%), but could have been slightly underestimated owing to the fact that no breakpoints have been set for some classes or compounds. On average resistance levels were unchanged in the past decade. Various antibiotics, approved in the past four decades, are available with high efficacy against BRD and SRD, but precise knowledge of the AMR situation remains essential for the targeted treatment of bacterial infections. Since in acute cases of disease antimicrobial therapy must be started immediately, the veterinary clinician has to rely on regional resistance data. Our data can be useful for veterinarians in selecting effective antimicrobial treatments. The findings of resistance to antibiotics with CLSI breakpoints, along with bimodal or multimodal MIC distribution for antibiotics without validated interpretation criteria, suggest the need for a continuous monitoring of the susceptibility patterns of isolates of respiratory pathogens to provide evidence-based guidance for antimicrobial therapy. In addition, the setting of the missing veterinary- and body site-specific breakpoints remains of high importance (Sweeney et al., 2018). Finally, this study also underlines the importance of responsible use of antibiotics when treating BRD or SRD.

## Data Availability

The original contributions presented in the study are included in the article/Supplementary material, further inquiries can be directed to the corresponding author/s.
