# Supplementary material for: Pan-European analysis shows stable, low antimicrobial resistance in most bovine and porcine respiratory tract pathogens
Source: Front Microbiol. 2026 Feb 23;17:1745115. doi: 10.3389/fmicb.2026.1745115 (PMC12967990; doi:10.3389/fmicb.2026.1745115)
Supplement: Supplementary file 1 [file Supplementary_file_1.docx]

|  |  |  |  |  |  |  |  |  |  |  |
| --- | --- | --- | --- | --- | --- | --- | --- | --- | --- | --- |

**Table S1**

Numbers of isolates of cattle and pig pathogens over eight countries from 2009 to 2020

| Cattle | Bacterium | Belgium | Czech Rep | Denmark | France | Germany | Italy | Netherlands | Poland | Spain | United Kingdom | All |
| --- | --- | --- | --- | --- | --- | --- | --- | --- | --- | --- | --- | --- |
|  | *Pasteurella multocida* | 95 | 51 | - | 51 | 74 | 52 | 43 | - | 20 | 24 | 410 |
|  | *Mannheimia haemolytica* | 105 | 33 | - | 57 | 42 | 37 | 44 | - | 21 | 21 | 360 |
|  |  |  |  |  |  |  |  |  |  |  |  |  |
| Pigs | *Pasteurella multocida* | 88 | - | 42 | 38 | 69 | - | 57 | 42 | 62 | 67 | 465 |
|  | *Actinobacillus pleuropneumoniae* | 108 | - | 50 | 36 | 54 | - | 73 | 30 | 62 | 31 | 444 |
|  | *Streptococcus suis* | 136 | - | 44 | 27 | 52 | - | 95 | 38 | 60 | 62 | 514 |
|  |  |  |  |  |  |  |  |  |  |  |  |  |

**Table S2**

Percentage resistance of cattle pathogens over eight countries from 2009 to 2020. The susceptibility to penicillin G and tildipirosin has not been assessed in the first survey 2009-2012. Consequently resistance percentages for these two antimicrobial agents have not been included in the statistical analysis.

|  |  |  |  |  |  |  |  |  |  |  |  |
| --- | --- | --- | --- | --- | --- | --- | --- | --- | --- | --- | --- |
| *Pasteurella multocida* | Antimicrobial agent | Belgium | Czech Rep | France | Germany | Italy | Netherlands | Spain | United Kingdom | All (n) | All (%) |
|  | Penicillin G | 1.1 | 0.0 | 0.0 | 1.4 | 0.0 | 0.0 | 0.0 | 0.0 | 2 | 0.5 |
|  | Ceftiofur | 0.0 | 0.0 | 0.0 | 0.0 | 0.0 | 0.0 | 0.0 | 0.0 | 0 | 0.0 |
|  | Danofloxacin | 2.1 | 7.8 | 3.9 | 1.4 | 1.9 | 0.0 | 5.2 | 0.0 | 11 | 2.7 |
|  | Enrofloxacin | 1.1 | 3.9 | 2.0 | 1.4 | 1.9 | 0.0 | 5.2 | 0.0 | 7 | 1.7 |
|  | Florfenicol | 4.2 | 0.0 | 2.0 | 1.4 | 0.0 | 2.3 | 0.0 | 0.0 | 7 | 1.7 |
|  | Gamithromycin | 11.6^a^ | 3.9 | 2.0 | 2.7^b^ | 1.9 | 2.3 | 5.2 | 0.0 | 19 | 4.6 |
|  | Tulathromycin | 8.4 | 3.9 | 2.0 | 1.4 | 3.8 | 0.0 | 5.2 | 4.2 | 16 | 3.9 |
|  | Tildipirosin | 11.6 | 2.0 | 2.0 | 2.7 | 0.0 | 0.0 | 0.0 | 0.0 | 15 | 3.7 |
|  | Spectinomycin | 26.3^a^ | 2.0^b,d^ | 3.9^b,d^ | 6.8^b,d^ | 3.9^b,d^ | 20.9^c^ | 5.2^b^ | 0.0^b,d^ | 45 | 11.0 |
|  | Tetracycline | 29.5^a^ | 11.8^b,d^ | 9.8^b,d^ | 13.5^b,d,e^ | 1.9^b,d,f^ | 34.9^c^ | 26.3 | 4.2^b,d^ | 71 | 17.3 |
|  |  |  |  |  |  |  |  |  |  |  |  |
| *Mannheimia haemolytica* | Penicillin G | 12.4 | 12.1 | 3.5 | 0.0 | 0.0 | 6.8 | 0.0 | 0.0 | 22 | 6.1 |
|  | Ceftiofur | 0.0 | 0.0 | 0.0 | 0.0 | 0.0 | 0.0 | 0.0 | 0.0 | 0 | 0.0 |
|  | Danofloxacin | 3.8^b^ | 6.1 | 1.8^b^ | 0.0^b^ | 0.0^b^ | 6.8 | 19.0^a^ | 4.8 | 15 | 4.0 |
|  | Enrofloxacin | 1.9 | 0.0 | 1.8 | 0.0 | 0.0 | 0.0 | 4.8 | 0.0 | 4 | 1.1 |
|  | Florfenicol | 1.0 | 0.0 | 0.0 | 0.0 | 0.0 | 4.5 | 0.0 | 0.0 | 3 | 0.8 |
|  | Gamithromycin | 2.9^b^ | 3.0 | 5.3 | 2.4 | 13.5^a^ | 2.3 | 0.0 | 0.0 | 14 | 3.9 |
|  | Tulathromycin | 1.0^b^ | 3.0 | 5.3 | 0.0^b^ | 13.5^a^ | 2.3 | 0.0 | 0.0 | 11 | 3.0 |
|  | Tildipirosin | 1.9 | 3.0 | 5.3 | 2.4 | 2.7 | 2.3 | 0.0 | 0.0 | 9 | 2.5 |
|  | Tilmicosin | 2.9 | 3.0 | 7.0 | 2.4 | 13.5 | 2.3 | 4.8 | 4.8 | 17 | 4.7 |
|  | Spectinomycin | 2.9 | 0.0 | 0.0 | 0.0 | 2.7 | 4.5 | 0.0 | 0.0 | 6 | 1.7 |
|  | Tetracycline | 22.9^a^ | 6.1^b^ | 12.2 | 19.0 | 16.2 | 18.2 | 14.3 | 0.0^b^ | 58 | 16.1 |
|  |  |  |  |  |  |  |  |  |  |  |  |

Different letters within a pair of letters (either a/b or c/d or e/f) indicate statistically significant differences among the eight countries of a given antimicrobial. The susceptibility to penicillin G and tildipirosin has not been assessed in the first survey 2009-2012; consequently resistance percentages for these two antimicrobials have not been included in the statistical analysis.

**Table S3**

Percentage resistance in pig pathogens over eight countries from 2009 to 2020. The susceptibility to penicillin G and tildipirosin has not been assessed in the first survey 2009-2012. Consequently resistance percentages for these two antimicrobial agents have not been included in the statistical analysis.

| *Pasteurella multocida* | Antimicrobial agent | Belgium | Denmark | France | Germany | Netherlands | Poland | Spain | United Kingdom | All (n) | All (%) |
| --- | --- | --- | --- | --- | --- | --- | --- | --- | --- | --- | --- |
|  | Amoxicillin | 1.1 | 11.9 | 0.0 | 0.0 | 5.3 | 0.0 | 1.6 | 3.0 | 12 | 2.6 |
|  | Penicillin G | 1.1 | 4.8 | 0.0 | 0.0 | 5.3 | 0.0 | 1.6 | 1.5 | 8 | 1.7 |
|  | Ceftiofur | 0.0 | 0.0 | 0.0 | 0.0 | 0.0 | 0.0 | 0.0 | 0.0 | 0 | 0.0 |
|  | Enrofloxacin | 0.0 | 0.0 | 0.0 | 0.0 | 0.0 | 4.8 | 0.0 | 1.5 | 3 | 0.6 |
|  | Florfenicol | 0.0 | 4.8 | 2.6 | 2.9 | 0.0 | 4.8 | 3.2 | 0.0 | 9 | 1.9 |
|  | Tulathromycin | 1.1 | 0.0 | 0.0 | 0.0 | 0.0 | 0.0 | 0.0 | 0.0 | 1 | 0.2 |
|  | Tildipirosin* | 1.1 | 2.4 | 0.0 | 2.9 | 0.0 | 0.0 | 0.0 | 1.5 | 5 | 1.1 |
|  | Tilmicosin | 2.3 | 0.0 | 0.0 | 0.0 | 0.0 | 0.0 | 0.0 | 3.0 | 4 | 0.9 |
|  | Tetracycline | 20.5^e^ | 0.0^b,d,f,h^ | 5.3^b,d,f^ | 2.9^b,d,f,h^ | 24.6^c^ | 31.0^a^ | 19.4^g^ | 7.5^b,d,f^ | 66 | 14.2 |
|  |  |  |  |  |  |  |  |  |  |  |  |
| *Actinobacillus pleuropneumoniae* | Amoxicillin | 4.6^b^ | 4.0^b^ | 2.8^b^ | 11.1^b,c^ | 0.0^b,d,f^ | 10.0^e^ | 27.4^a,d^ | 3.2^b^ | 35 | 7.9 |
|  | Ceftiofur | 0.0 | 0.0 | 0.0 | 0.0 | 0.0 | 0.0 | 0.0 | 0.0 | 0 | 0.0 |
|  | Enrofloxacin | 0.0^b^ | 0.0 | 2.8 | 0.0 | 0.0 | 0.0 | 4.8^a^ | 0.0 | 4 | 0.9 |
|  | Florfenicol | 0.0 | 0.0 | 0.0 | 0.0 | 0.0 | 3.3 | 0.0 | 0.0 | 1 | 0.2 |
|  | Tulathromycin | 0.0 | 2.0 | 0.0 | 0.0 | 0.0 | 0.0 | 0.0 | 0.0 | 1 | 0.2 |
|  | Tildipirosin* | 0.0 | 4.0 | 0.0 | 0.0 | 1.4 | 0.0 | 4.8 | 0.0 | 6 | 1.4 |
|  | Tilmicosin | 7.4 | 12.0 | 0.0^b,d,f^ | 16.7^a^ | 13.7^e^ | 0.0^b^ | 14.5^c^ | 0.0^b^ | 42 | 9.5 |
|  | Tiamulin | 1.9 | 0.0 | 0.0 | 1.9 | 2.7 | 0.0 | 1.6 | 3.2 | 7 | 1.6 |
|  | Tetracycline | 11.1^b,d^ | 24.0^b,d,e^ | 13.9^b,d^ | 9.3^b,d^ | 5.5^b,d,f,h^ | 20.0^b,d,g^ | 45.2^c,f,h^ | 64.5^a,f,h^ | 92 | 20.7 |
|  |  |  |  |  |  |  |  |  |  |  |  |
| *Streptococcus suis* | Amoxicillin | 2.9 | 0.0 | 0.0 | 0.0 | 1.1 | 0.0 | 5.0 | 1.6 | 9 | 1.8 |
|  | Penicillin G | 3.7 | 0.0 | 0.0 | 0.0 | 2.1 | 0.0 | 10.0 | 3.2 | 15 | 2.9 |
|  | Ceftiofur | 2.9 | 0.0 | 0.0 | 0.0 | 1.1^b^ | 0.0 | 8.3^a^ | 1.6 | 11 | 2.1 |
|  | Enrofloxacin | 1.5^b^ | 0.0 | 0.0 | 0.0 | 0.0^b^ | 0.0 | 8.3^a^ | 4.8 | 10 | 1.9 |
|  | Florfenicol | 0.0 | 0.0 | 0.0 | 0.0 | 2.1 | 0.0 | 3.3 | 1.6 | 5 | 1.0 |
|  | Tetracycline | 87.5^b^ | 70.5^a^ | 81.5 | 86.5 | 84.2 | 84.2 | 93.3^b,d^ | 79.0^c^ | 434 | 84.4 |
|  |  |  |  |  |  |  |  |  |  |  |  |

*For Tildipirosin only susceptible breakpoints have been set (CLSI, 2024). In this analysis non-susceptible isolates were considered as tildipirosin-resistant isolates.

Different letters within a pair of letters (either a/b or c/d or e/f or g/h) indicate statistically significant differences among the eight countries of a given antimicrobial. The susceptibility to penicillin G and tildipirosin has not been assessed in the first survey 2009-2012; consequently resistance percentages for these two antimicrobials have not been included in the statistical analysis.

**Table S4**

MIC distribution frequencies of *Pasteurella multocida* cattle isolates from the EU 2019 – 2020

|  | *P. multocida* (*n* = 145) | | | | | | | | | | | | | | | | | | | | | | | | |
| --- | --- | --- | --- | --- | --- | --- | --- | --- | --- | --- | --- | --- | --- | --- | --- | --- | --- | --- | --- | --- | --- | --- | --- | --- | --- |
| Antimicrobial Agent | MIC values (µg ml^-1^) | | | | | | | | | | | | | | | | | | |  | MIC_50_  (µg ml^-1^) | MIC_90_  (µg ml^-1^) | S  (%) | I  (%) | R  (%) |
|  | 0.002 | 0.004 | 0.008 | 0.015 | 0.03 | 0.06 | 0.12 | 0.25 | 0.5 | 1 | 2 | 4 | 8 | 16 | 32 | 64 | 128 | 256 | 512 | >512 |  |  |  |  |  |
| Amoxicillin |  |  |  |  | 1 |  | 40 | 97 | 3 | 3 |  |  |  |  |  | 1 |  |  |  |  | 0.25 | 0.25 | - | - | - |
| Amoxicillin clavulanic acid^*^ |  |  |  |  |  | 1 | 30 | 105 | 7 | 2 |  |  |  |  |  |  |  |  |  |  | 0.25 | 0.25 | - | - | - |
| Penicillin G |  |  |  |  | 2 | 91 | 46 | 4 | 1 |  |  |  |  |  | 1 |  |  |  |  |  | 0.06 | 0.12 | 98.6 | 0.7 | 0.7 |
| Penicillin/streptomycin^**^ |  |  |  |  | 1 | 97 | 38 | 7 | 1 |  |  |  | 1 |  |  |  |  |  |  |  | 0.06 | 0.12 | - | - | - |
| Cefquinome |  |  |  | 46 | 67 | 29 | 3 |  |  |  |  |  |  |  |  |  |  |  |  |  | 0.03 | 0.06 | - | - | - |
| Ceftiofur | 45 | 64 | 13 | 11 | 4 | 1 | 4 | 3 |  |  |  |  |  |  |  |  |  |  |  |  | 0.004 | 0.03 | 100.0 | 0.0 | 0.0 |
| Colistin |  |  |  |  |  | 1 | 8 | 65 | 35 | 21 | 12 | 2 |  | 1 |  |  |  |  |  |  | 0.25 | 2 | - | - | - |
| Danofloxacin |  | 3 | 33 | 46 | 48 | 5 | 5 | 1 |  | 1 | 2 |  |  | 1 |  |  |  |  |  |  | 0.015 | 0.06 | 97.2 | 0.0 | 2.8 |
| Enrofloxacin |  | 11 | 46 | 51 | 26 | 2 | 5 |  | 1 | 1 | 1 |  |  |  | 1 |  |  |  |  |  | 0.015 | 0.03 | 97.2 | 1.4 | 1.4 |
| Marbofloxacin |  | 2 | 7 | 60 | 60 | 5 | 3 | 4 | 1 | 1 | 1 |  |  | 1 |  |  |  |  |  |  | 0.03 | 0.06 | - | - | - |
| Florfenicol |  |  |  |  |  |  | 2 | 11 | 121 | 8 |  |  |  | 2 | 1 |  |  |  |  |  | 0.5 | 1 | 97.9 | 0.0 | 2.1 |
| Thiamphenicol |  |  |  |  |  |  |  | 1 | 39 | 93 | 1 | 1 |  | 1 | 2 | 7 |  |  |  |  | 1 | 1 | - | - | - |
| Doxycycline |  |  |  |  |  | 1 | 46 | 38 | 20 | 30 | 4 | 6 |  |  |  |  |  |  |  |  | 0.25 | 1 | - | - | - |
| Tetracycline |  |  |  |  |  |  | 4 | 66 | 19 | 10 | 1 | 2 | 20 | 14 | 4 | 5 |  |  |  |  | 0.5 | 16 | 69.0 | 1.4 | 29.7 |
| Gamithromycin |  |  |  |  |  |  | 4 | 6 | 40 | 61 | 14 | 4 |  | 1 | 2 | 2 |  | 11 |  |  | 1 | 32 | 89.0 | 0.0 | 11.0 |
| Tulathromycin |  |  |  |  |  |  | 1 | 5 | 34 | 61 | 18 | 5 | 3 | 5 | 1 | 1 | 7 | 4 |  |  | 1 | 16 | 91.0 | 0.7 | 8.3 |
| Tildipirosin |  |  |  |  |  |  | 1 | 3 | 15 | 54 | 40 | 13 | 3 | 2 | 1 | 2 |  | 11 |  |  | 1 | 16 | 89.0 | 1.4 | 9.7 |
| Tilmicosin |  |  |  |  |  |  |  | 2 | 2 | 8 | 31 | 55 | 29 | 4 | 2 |  | 1 | 11 |  |  | 4 | 16 | - | - | - |
| Tylosin |  |  |  |  |  |  |  |  | 1 | 2 | 2 | 9 | 16 | 39 | 48 | 13 | 3 | 1 | 11 |  | 32 | 128 | - | - | - |
| Tiamulin |  |  |  |  |  |  | 2 | 1 |  | 3 | 7 | 20 | 48 | 48 | 7 | 9 |  |  |  |  | 8 | 32 | - | - | - |
| Lincomycin |  |  |  |  |  |  |  |  |  |  |  |  | 1 | 14 | 103 | 15 |  | 12 |  |  | 32 | 64 | - | - | - |
| Lincomycin/spectinomycin |  |  |  |  |  |  |  |  |  |  | 1 | 14 | 89 | 9 | 20 | 3 | 1 | 7 | 1 |  | 8 | 32 | - | - | - |
| Spectinomycin |  |  |  |  |  |  |  |  |  |  |  | 1 | 2 | 42 | 65 | 3 |  |  | 1 | 31 | 32 | >512 | 75.9 | 2.1 | 22.1 |
| Trimetho/sulfamethoxazole^***^ |  |  |  | 2 | 26 | 56 | 20 | 7 | 6 | 23 | 4 |  | 1 |  |  |  |  |  |  |  | 0.06 | 1 | - | - | - |

The dilution ranges tested are those contained in the white area. Values above this range indicate MIC values higher than the highest concentration within the range. Values corresponding to the lowest concentration tested indicate MIC values lower or equal to the lowest concentration within the range. When available, susceptible and resistance breakpoints are indicated in vertical green and red lines. A dash indicates that no figure could be calculated because no CLSI interpretive criteria are available.

*Concentration for amoxicillin given, tested with clavulanic acid in a concentration ratio 2:1.

**Concentration of penicillin is given, tested in a concentration ratio of 1:2 (penicillin/streptomycin).

***Concentration of trimethoprim is given, tested in a concentration ratio of 1:19 (trimethoprim/sulfamethoxazole).

**Table S5**

MIC distribution frequencies of *Mannheimia haemolytica* cattle isolates from the EU 2019 – 2020

|  | *M. haemolytica* (*n* = 133) | | | | | | | | | | | | | | | | | | | | | | | | |
| --- | --- | --- | --- | --- | --- | --- | --- | --- | --- | --- | --- | --- | --- | --- | --- | --- | --- | --- | --- | --- | --- | --- | --- | --- | --- |
| Antimicrobial Agent | MIC values (µg ml^-1^) | | | | | | | | | | | | | | | | | | |  | MIC_50_  (µg ml^-1^) | MIC_90_  (µg ml^-1^) | S  (%) | I  (%) | R  (%) |
|  | 0.002 | 0.004 | 0.008 | 0.015 | 0.03 | 0.06 | 0.12 | 0.25 | 0.5 | 1 | 2 | 4 | 8 | 16 | 32 | 64 | 128 | 256 | 512 | >512 |  |  |  |  |  |
| Amoxicillin |  |  |  | 1 | 3 | 6 | 61 | 38 | 7 | 2 |  | 1 |  | 5 | 3 | 6 |  |  |  |  | 0.12 | 16 | - | - | - |
| Amoxicillin clavulanic acid^*^ |  |  |  |  | 2 | 2 | 68 | 45 | 12 | 3 | 1 |  |  |  |  |  |  |  |  |  | 0.25 | 0.5 | - | - | - |
| Penicillin G |  |  |  |  | 2 | 14 | 59 | 26 | 12 | 4 | 1 | 1 |  | 1 | 13 |  |  |  |  |  | 0.12 | 16 | 75.9 | 9.0 | 15.0 |
| Penicillin/streptomycin^**^ |  |  |  |  | 2 | 6 | 62 | 32 | 12 | 4 | 1 | 4 | 5 |  | 5 |  |  |  |  |  | 0.12 | 4 | - | - | - |
| Cefquinome |  | 4 | 16 | 56 | 37 | 15 | 5 |  |  |  |  |  |  |  |  |  |  |  |  |  | 0.015 | 0.06 | - | - | - |
| Ceftiofur | 4 | 14 | 47 | 45 | 15 | 7 | 1 |  |  |  |  |  |  |  |  |  |  |  |  |  | 0.015 | 0.03 | 100.0 | 0.0 | 0.0 |
| Colistin |  |  |  |  |  | 2 | 17 | 109 | 1 | 3 | 1 |  |  |  |  |  |  |  |  |  | 0.25 | 0.25 | - | - | - |
| Danofloxacin |  |  | 2 | 5 | 65 | 22 | 5 | 12 | 19 | 1 | 1 |  |  | 1 |  |  |  |  |  |  | 0.03 | 0.5 | 83.5 | 14.3 | 2.3 |
| Enrofloxacin |  | 1 | 1 | 6 | 68 | 18 | 5 | 6 | 25 | 2 |  |  |  | 1 |  |  |  |  |  |  | 0.03 | 0.5 | 79.0 | 20.3 | 0.8 |
| Marbofloxacin |  |  | 1 | 13 | 75 | 8 | 1 | 29 | 4 | 1 |  |  | 1 |  |  |  |  |  |  |  | 0.03 | 0.25 | - | - | - |
| Florfenicol |  |  |  |  |  |  | 1 | 2 | 9 | 102 | 16 |  | 1 | 1 | 1 |  |  |  |  |  | 1 | 2 | 92.7 | 0.0 | 2.3 |
| Thiamphenicol |  |  |  |  |  |  |  | 1 | 1 | 52 | 53 |  |  | 3 | 1 | 22 |  |  |  |  | 2 | >32 | - | - | - |
| Doxycycline |  |  |  |  |  |  | 1 | 29 | 54 | 22 | 12 | 10 | 3 | 1 | 1 |  |  |  |  |  | 0.5 | 4 | - | - | - |
| Tetracycline |  |  |  |  |  |  | 2 | 15 | 77 | 11 | 1 | 1 | 1 | 14 | 11 |  |  |  |  |  | 0.5 | 16 | 79.7 | 0.8 | 19.6 |
| Gamithromycin |  |  |  |  |  | 1 | 1 | 1 | 8 | 81 | 36 | 2 |  | 1 |  |  | 1 | 1 |  |  | 1 | 2 | 97.7 | 0.0 | 2.3 |
| Tulathromycin |  |  |  |  |  |  |  | 2 | 6 | 21 | 62 | 37 | 3 |  |  |  | 1 | 1 |  |  | 2 | 4 | 98.5 | 0.0 | 1.5 |
| Tildipirosin |  |  |  |  |  | 1 |  | 1 | 29 | 62 | 30 | 4 | 3 | 1 |  |  | 1 | 1 |  |  | 1 | 2 | 95.5 | 2.3 | 2.3 |
| Tilmicosin |  |  |  |  |  |  | 1 | 1 |  | 1 | 25 | 67 | 32 | 3 | 1 |  |  | 2 |  |  | 4 | 8 | 95.5 | 2.3 | 2.3 |
| Tylosin |  |  |  |  |  |  | 1 |  | 1 |  | 1 |  | 6 | 8 | 49 | 59 | 6 |  | 2 |  | 64 | 64 | - | - | - |
| Tiamulin |  |  |  |  |  |  |  | 1 |  | 1 | 3 | 20 | 61 | 42 | 5 |  |  |  |  |  | 8 | 16 | - | - | - |
| Lincomycin |  |  |  |  |  |  |  |  |  |  |  | 2 |  | 3 | 20 | 77 | 27 | 4 |  |  | 64 | 128 | - | - | - |
| Lincomycin/spectinomycin |  |  |  |  |  |  |  |  |  | 1 | 1 | 6 | 84 | 39 | 1 |  |  |  | 1 |  | 8 | 16 | - | - | - |
| Spectinomycin |  |  |  |  |  |  |  |  |  |  |  | 1 | 2 | 40 | 86 | 1 |  |  | 2 | 1 | 32 | 32 | 97.0 | 0.8 | 2.3 |
| Trimetho/sulfamethoxazole^b***^ |  | 1 | 1 | 4 | 84 | 19 | 5 | 8 | 6 | 4 |  |  |  |  | 1 |  |  |  |  |  | 0.03 | 0.25 | - | - | - |

The dilution ranges tested are those contained in the white area. Values above this range indicate MIC values higher than the highest concentration within the range. Values corresponding to the lowest concentration tested indicate MIC values lower or equal to the lowest concentration within the range. When available, susceptible and resistance breakpoints are indicated in vertical green and red lines. A dash indicates that no figure could be calculated because no CLSI interpretive criteria are available.

*Concentration for amoxicillin given, tested with clavulanic acid in a concentration ratio 2:1.

**Concentration of penicillin is given, tested in a concentration ratio of 1:2 (penicillin/streptomycin).

***Concentration of trimethoprim is given, tested in a concentration ratio of 1:19 (trimethoprim/sulfamethoxazole).

**Table S6**

MIC distribution frequencies of *Histophilus somni* cattle isolates from the EU 2019 – 2020

| *H. somni* (*n* = 29) | | | | | | | | | | | | | | | | | | | | | | | | |
| --- | --- | --- | --- | --- | --- | --- | --- | --- | --- | --- | --- | --- | --- | --- | --- | --- | --- | --- | --- | --- | --- | --- | --- | --- |
| Antimicrobial Agent | MIC values (µg ml^-1^) | | | | | | | | | | | | | | | | | | | MIC_50_  (µg ml^-1^) | MIC_90_  (µg ml^-1^) | S  (%) | I  (%) | R  (%) |
|  | 0.002 | 0.004 | 0.008 | 0.015 | 0.03 | 0.06 | 0.12 | 0.25 | 0.5 | 1 | 2 | 4 | 8 | 16 | 32 | 64 | 128 | 256 | 512 |  |  |  |  |  |
| Amoxicillin |  | 8 | 1 | 2 | 13 | 2 | 2 | 1 |  |  |  |  |  |  |  |  |  |  |  | 0.03 | 0.12 | - | - | - |
| Amoxicillin clavulanic acid^*^ |  |  |  |  | 21 | 6 | 1 | 1 |  |  |  |  |  |  |  |  |  |  |  | ≤0.03 | 0.06 | - | - | - |
| Penicillin G |  | 10 | 12 | 5 | 1 | 1 |  |  |  |  |  |  |  |  |  |  |  |  |  | 0.008 | 0.015 | 100.0 | 0.0 | 0.0 |
| Penicillin/streptomycin^**^ |  | 6 | 15 | 6 |  | 2 |  |  |  |  |  |  |  |  |  |  |  |  |  | 0.008 | 0.015 | - | - | - |
| Cefquinome |  | 17 | 11 | 1 |  |  |  |  |  |  |  |  |  |  |  |  |  |  |  | ≤0.004 | 0.008 | - | - | - |
| Ceftiofur | 22 | 7 |  |  |  |  |  |  |  |  |  |  |  |  |  |  |  |  |  | ≤0.002 | 0.004 | 100.0 | 0.0 | 0.0 |
| Colistin |  |  |  |  |  | 13 | 5 | 4 | 5 | 1 | 1 |  |  |  |  |  |  |  |  | 0.12 | 0.5 | - | - | - |
| Danofloxacin |  | 6 |  | 1 | 2 | 17 | 3 |  |  |  |  |  |  |  |  |  |  |  |  | 0.06 | 0.06 | - | - | - |
| Enrofloxacin |  | 6 |  | 3 | 17 | 3 |  |  |  |  |  |  |  |  |  |  |  |  |  | 0.03 | 0.06 | 100.0 | 0.0 | 0.0 |
| Marbofloxacin |  | 6 |  | 2 | 11 | 10 |  |  |  |  |  |  |  |  |  |  |  |  |  | 0.03 | 0.06 | - | - | - |
| Florfenicol |  |  |  |  |  |  | 15 | 14 |  |  |  |  |  |  |  |  |  |  |  | ≤0.12 | 0.25 | 100.0 | 0.0 | 0.0 |
| Thiamphenicol |  |  |  |  |  |  | 11 | 14 | 4 |  |  |  |  |  |  |  |  |  |  | 0.25 | 0.5 | - | - | - |
| Doxycycline |  |  |  |  |  | 15 | 10 | 3 | 1 |  |  |  |  |  |  |  |  |  |  | ≤0.06 | 0.25 | - | - | - |
| Tetracycline |  |  |  |  |  | 8 | 13 | 7 |  |  |  |  | 1 |  |  |  |  |  |  | 0.12 | 0.25 | 96.6 | 0.0 | 3.5 |
| Gamithromycin |  |  |  |  |  | 6 | 10 | 10 | 2 | 1 |  |  |  |  |  |  |  |  |  | 0.12 | 0.5 | 100.0 | 0.0 | 0.0 |
| Tulathromycin |  |  |  |  |  |  | 6 | 1 | 3 | 4 | 10 | 4 | 1 |  |  |  |  |  |  | 2 | 4 | 100.0 | 0.0 | 0.0 |
| Tildipirosin |  |  |  | 2 |  | 1 |  |  | 1 | 13 | 6 | 6 |  |  |  |  |  |  |  | 1 | 4 | 100.0 | 0.0 | 0.0 |
| Tilmicosin |  |  |  |  |  |  | 6 | 3 | 2 | 5 | 9 | 4 |  |  |  |  |  |  |  | 1 | 4 | - | - | - |
| Tylosin |  |  |  |  |  |  | 5 | 3 | 2 | 4 | 12 | 2 | 1 |  |  |  |  |  |  | 2 | 4 | - | - | - |
| Tiamulin |  |  |  |  |  |  | 9 | 6 | 12 | 2 |  |  |  |  |  |  |  |  |  | 0.25 | 0.5 | - | - | - |
| Lincomycin |  |  |  |  |  |  |  |  |  |  |  | 28 | 1 |  |  |  |  |  |  | ≤4 | ≤4 | - | - | - |
| Lincomycin/spectinomycin |  |  |  |  |  |  |  |  |  | 16 | 8 | 5 |  |  |  |  |  |  |  | ≤1 | 4 | - | - | - |
| Spectinomycin |  |  |  |  |  |  |  |  |  |  | 6 | 1 | 13 | 8 |  |  |  |  | 1 | 8 | 16 | 96.6 | 0.0 | 3.5 |
| Trimetho/sulfamethoxazole^***^ |  | 3 | 1 |  |  | 8 | 16 | 1 |  |  |  |  |  |  |  |  |  |  |  | 0.12 | 0.12 | - | - | - |

The dilution ranges tested are those contained in the white area. Values above this range indicate MIC values higher than the highest concentration within the range. Values corresponding to the lowest concentration tested indicate MIC values lower or equal to the lowest concentration within the range. When available, susceptible and resistance breakpoints are indicated in vertical green and red lines. A dash indicates that no figure could be calculated because no CLSI interpretive criteria are available.

*Concentration for amoxicillin given, tested with clavulanic acid in a concentration ratio 2:1.

**Concentration of penicillin is given, tested in a concentration ratio of 1:2 (penicillin/streptomycin).

***Concentration of trimethoprim is given, tested in a concentration ratio of 1:19 (trimethoprim/sulfamethoxazole).

**Table S7**

MIC distribution frequencies of *Pasteurella multocida* pig isolates from the EU 2019 – 2020

|  | *P. multocida* (*n* = 149) | | | | | | | | | | | | | | | | | | | | | | | | |
| --- | --- | --- | --- | --- | --- | --- | --- | --- | --- | --- | --- | --- | --- | --- | --- | --- | --- | --- | --- | --- | --- | --- | --- | --- | --- |
| Antimicrobial Agent | MIC values (µg ml^-1^) | | | | | | | | | | | | | | | | | | | | MIC_50_  (µg ml^-1^) | MIC_90_  (µg ml^-1^) | S  (%) | I  (%) | R  (%) |
|  | 0.002 | 0.004 | 0.008 | 0.015 | 0.03 | 0.06 | 0.12 | 0.25 | 0.5 | 1 | 2 | 4 | 8 | 16 | 32 | 64 | 128 | 256 | 512 | >512 |  |  |  |  |  |
| Amoxicillin |  |  |  |  |  | 3 | 68 | 74 | 2 |  |  |  |  |  | 1 |  | 1 |  |  |  | 0.25 | 0. 25 | 98.7 | 0.0 | 1.3 |
| Amoxicillin/clavulanic acid^*^ |  |  |  |  |  |  | 67 | 80 | 2 |  |  |  |  |  |  |  |  |  |  |  | 0.25 | 0.25 | - | - | - |
| Penicillin G |  |  |  |  | 6 | 117 | 24 |  |  |  |  |  |  |  | 2 |  |  |  |  |  | 0.06 | 0.12 | 98.7 | 0.0 | 1.3 |
| Penicillin/streptomycin^**^ |  |  |  |  | 9 | 115 | 23 |  |  |  |  |  | 1 |  | 1 |  |  |  |  |  | 0.06 | 0.12 | - | - | - |
| Cefquinome |  | 1 |  | 35 | 70 | 14 | 1 | 2 | 6 | 12 | 7 | 1 |  |  |  |  |  |  |  |  | 0.03 | 1 | - | - | - |
| Ceftiofur | 55 | 48 | 10 | 4 | 2 | 9 | 11 | 7 |  | 3 |  |  |  |  |  |  |  |  |  |  | 0.004 | 0.12 | 100.0 | 0.0 | 0.0 |
| Colistin |  |  |  |  |  | 5 | 4 | 15 | 44 | 59 | 19 | 1 | 2 |  |  |  |  |  |  |  | 1 | 2 | - | - | - |
| Danofloxacin |  | 1 | 17 | 86 | 33 | 3 | 4 | 3 |  |  | 2 |  |  |  |  |  |  |  |  |  | 0.015 | 0.03 | - | - | - |
| Enrofloxacin |  | 2 | 61 | 58 | 18 | 3 | 4 | 1 |  | 2 |  |  |  |  |  |  |  |  |  |  | 0.015 | 0.03 | 98.7 | 0.0 | 1.3 |
| Marbofloxacin |  | 1 | 4 | 77 | 50 | 7 | 3 | 5 |  | 2 |  |  |  |  |  |  |  |  |  |  | 0.015 | 0.06 | - | - | - |
| Florfenicol |  |  |  |  |  |  | 1 | 12 | 133 | 2 |  |  |  |  | 1 |  |  |  |  |  | 0.5 | 0.5 | 99.3 | 0.0 | 0.7 |
| Thiamphenicol |  |  |  |  |  |  |  | 1 | 58 | 88 | 1 |  |  |  |  | 1 |  |  |  |  | 1 | 1 | - | - | - |
| Doxycycline |  |  |  |  |  | 3 | 52 | 56 | 17 | 13 | 3 | 5 |  |  |  |  |  |  |  |  | 0.25 | 1 | - | - | - |
| Tetracycline |  |  |  |  |  |  | 3 | 68 | 41 | 20 | 4 | 1 | 3 | 2 | 5 | 2 |  |  |  |  | 0.5 | 2 | 75.2 | 13.4 | 11.4 |
| Gamithromycin |  |  |  |  |  |  |  | 9 | 49 | 54 | 34 | 2 |  |  |  |  |  | 1 |  |  | 1 | 2 | - | - | - |
| Tulathromycin |  |  |  |  |  |  |  | 17 | 53 | 62 | 14 | 1 | 1 |  |  | 1 |  |  |  |  | 1 | 2 | 99.3 | 0.0 | 0.7 |
| Tildipirosin |  |  |  |  |  |  |  | 4 | 31 | 48 | 51 | 12 | 2 |  |  |  |  | 1 |  |  | 1 | 4 | 98.0^†^ | - | - |
| Tilmicosin |  |  |  |  |  |  |  |  | 1 | 14 | 41 | 46 | 42 | 4 |  |  |  | 1 |  |  | 4 | 8 | 99.3 | - | 0.7 |
| Tylosin |  |  |  |  |  |  |  |  |  |  | 1 | 2 | 7 | 40 | 82 | 16 |  | 1 |  |  | 32 | 64 | - | - | - |
| Tiamulin |  |  |  |  |  |  |  | 1 |  |  | 2 | 14 | 56 | 50 | 26 |  |  |  |  |  | 16 | 32 | - | - | - |
| Lincomycin |  |  |  |  |  |  |  |  |  |  |  |  |  | 10 | 117 | 21 |  | 1 |  |  | 32 | 64 | - | - | - |
| Lincomycin/spectinomycin |  |  |  |  |  |  |  |  |  |  |  | 5 | 108 | 28 | 6 | 1 | 1 |  |  |  | 8 | 16 | - | - | - |
| Spectinomycin |  |  |  |  |  |  |  |  |  |  |  |  | 2 | 39 | 87 | 13 |  |  |  | 8 | 32 | 64 | - | - | - |
| Trimetho/sulfamethoxazole^***^ |  |  |  | 1 | 27 | 56 | 30 | 8 | 7 | 7 | 2 |  |  | 1 | 10 |  |  |  |  |  | 0.06 | 1 | - | - | - |

The dilution ranges tested are those contained in the white area. Values above this range indicate MIC values higher than the highest concentration within the range. Values corresponding to the lowest concentration tested indicate MIC values lower or equal to the lowest concentration within the range. When available, susceptible and resistance breakpoints are indicated in vertical green and red lines. A dash indicates that no figure could be calculated because no CLSI interpretive criteria are available. ^†^Three isolates were non-susceptible to tildipirosin.

*Concentration for amoxicillin given, tested with clavulanic acid in a concentration ratio 2:1.

**Concentration of penicillin is given, tested with streptomycin in a concentration ratio 1:2.

***Concentration for trimethoprim given, tested with sulfamethoxazole in a concentration ratio 1:19.

**Table S8**

MIC distribution frequencies of *Actinobacillus pleuropneumoniae* pig isolates from the EU 2019 – 2020.

| *A. pleuropneumoniae* (*n* = 151) | | | | | | | | | | | | | | | | | | | | | | | | |
| --- | --- | --- | --- | --- | --- | --- | --- | --- | --- | --- | --- | --- | --- | --- | --- | --- | --- | --- | --- | --- | --- | --- | --- | --- |
| Antimicrobial Agent | MIC values (µg ml^-1^) | | | | | | | | | | | | | | | | | | | MIC_50_  (µg ml^-1^) | MIC_90_  ((µg ml^-1^) | S  (%) | I  (%) | R  (%) |
|  | 0.002 | 0.004 | 0.008 | 0.015 | 0.03 | 0.06 | 0.12 | 0.25 | 0.5 | 1 | 2 | 4 | 8 | 16 | 32 | 64 | 128 | 256 | 512 |  |  |  |  |  |
| Amoxicillin |  |  |  |  |  | 1 | 3 | 55 | 83 | 1 |  |  |  | 5 |  | 2 | 1 |  |  | 0.5 | 0.5 | 94.0 | 0.7 | 5.3 |
| Amoxicillin/clavulanic acid^*^ |  |  |  |  | 1 | 1 | 3 | 51 | 94 | 1 |  |  |  |  |  |  |  |  |  | 0.5 | 0.5 | - | - | - |
| Penicillin G |  |  |  |  | 1 | 2 | 12 | 59 | 66 | 3 |  |  |  |  | 8 |  |  |  |  | 0.5 | 0.5 | - | - | - |
| Penicillin/streptomycin^**^ |  |  |  |  | 1 | 3 | 12 | 55 | 68 | 4 |  |  |  |  | 8 |  |  |  |  | 0.5 | 0.5 | - | - | - |
| Cefquinome |  |  | 21 | 99 | 29 | 1 |  |  | 1 |  |  |  |  |  |  |  |  |  |  | 0.015 | 0.03 | - | - | - |
| Ceftiofur | 1 |  | 2 | 81 | 63 | 3 |  |  |  |  | 1 |  |  |  |  |  |  |  |  | 0.015 | 0.03 | 100.0 | 0.0 | 0.0 |
| Colistin |  |  |  |  |  | 1 | 2 | 30 | 59 | 56 | 2 |  | 1 |  |  |  |  |  |  | 0.5 | 1 | - | - | - |
| Danofloxacin |  |  |  | 4 | 54 | 87 | 3 | 2 |  | 1 |  |  |  |  |  |  |  |  |  | 0.06 | 0.06 | - | - | - |
| Enrofloxacin |  |  | 1 | 17 | 106 | 24 |  | 2 | 1 |  |  |  |  |  |  |  |  |  |  | 0.03 | 0.06 | 99.3 | 0.7 | 0.0 |
| Marbofloxacin |  |  |  | 23 | 120 | 5 |  | 2 |  | 1 |  |  |  |  |  |  |  |  |  | 0.03 | 0.03 | - | - | - |
| Florfenicol |  |  |  |  |  |  |  | 11 | 135 | 2 |  | 1 | 1 |  | 1 |  |  |  |  | 0.5 | 0.5 | 98.0 | 0.7 | 1.3 |
| Thiamphenicol |  |  |  |  |  |  |  | 1 | 46 | 98 | 2 | 1 | 2 |  |  | 1 |  |  |  | 1 | 1 | - | - | - |
| Doxycycline |  |  |  |  |  |  |  | 3 | 109 | 20 | 12 | 6 | 1 |  |  |  |  |  |  | 0.5 | 2 | - | - | - |
| Tetracycline |  |  |  |  |  |  |  | 12 | 115 | 3 |  | 1 | 9 | 8 | 3 |  |  |  |  | 0.5 | 8 | 84.1 | 2.0 | 13.9 |
| Gamithromycin |  |  |  |  |  |  |  |  | 1 | 2 | 114 | 24 | 10 |  |  |  |  |  |  | 2 | 4 | - | - | - |
| Tulathromycin |  |  |  |  |  |  |  |  |  |  |  | 5 | 25 | 70 | 47 | 4 |  |  |  | 16 | 32 | 100.0 | - | - |
| Tildipirosin |  |  |  |  |  |  |  |  |  | 1 | 4 | 16 | 63 | 61 | 5 | 1 |  |  |  | 8 | 16 | 96.0† | - | - |
| Tilmicosin |  |  |  |  |  |  |  |  |  |  |  | 4 | 37 | 93 | 17 |  |  |  |  | 16 | 32 | 88.7 | - | 11.3 |
| Tylosin |  |  |  |  |  |  |  |  |  | 1 |  | 2 | 6 | 3 | 68 | 69 | 2 |  |  | 32 | 64 | - | - | - |
| Tiamulin |  |  |  |  |  |  |  | 1 | 1 | 2 | 1 | 2 | 77 | 64 | 3 |  |  |  |  | 8 | 16 | 98.0 | - | 2.0 |
| Lincomycin |  |  |  |  |  |  |  |  |  |  |  |  | 4 | 15 | 77 | 54 |  | 1 |  | 32 | 64 | - | - | - |
| Lincomycin/spectinomycin |  |  |  |  |  |  |  |  |  |  |  |  | 6 | 109 | 36 |  |  |  |  | 16 | 32 | - | - | - |
| Spectinomycin |  |  |  |  |  |  |  |  |  |  |  |  |  |  | 4 | 97 | 48 | 2 |  | 64 | 128 | - | - | - |
| Trimetho/sulfamethoxazole^***^ |  |  | 1 | 9 | 24 | 45 | 56 | 2 | 4 | 9 | 1 |  |  |  |  |  |  |  |  | 0.06 | 0.25 | - | - | - |

The dilution ranges tested are those contained in the white area. Values above this range indicate MIC values higher than the highest concentration within the range. Values corresponding to the lowest concentration tested indicate MIC values lower or equal to the lowest concentration within the range. When available, susceptible and resistance breakpoints are indicated in vertical green and red lines. A dash indicates that no figure could be calculated because no CLSI interpretive criteria are available. †Five isolates were non-susceptible to tildipirosin.

*Concentration for amoxicillin given, tested with clavulanic acid in a concentration ratio 2:1.

**Concentration of penicillin is given, tested with streptomycin in a concentration ratio 1:2.

***Concentration for trimethoprim given, tested with sulfamethoxazole in a concentration ratio 1:19.

**Table S9**

MIC distribution frequencies of *Streptococcus suis* pig isolates from the EU 2019 – 2020.

| *S. suis* (*n* = 232) | | | | | | | | | | | | | | | | | | | | | | | |
| --- | --- | --- | --- | --- | --- | --- | --- | --- | --- | --- | --- | --- | --- | --- | --- | --- | --- | --- | --- | --- | --- | --- | --- |
| Antimicrobial Agent | MIC values (µg ml^-1^) | | | | | | | | | | | | | | | | | | MIC_50_  (µg ml^-1^) | MIC_90_  (µg ml^-1^) | S  (%) | I  (%) | R  (%) |
|  | 0.008 | 0.015 | 0.03 | 0.06 | 0.12 | 0.25 | 0.5 | 1 | 2 | 4 | 8 | 16 | 32 | 64 | 128 | 256 | 512 | >512 |  |  |  |  |  |
| Amoxicillin | 5 | 132 | 75 | 7 | 5 | 1 | 2 | 1 | 3 |  | 1 |  |  |  |  |  |  |  | 0.015 | 0.03 | 97.8 | 0.4 | 1.7 |
| Amoxicillin/clavulanic acid^*^ |  |  | 212 | 8 | 5 | 1 | 1 | 2 | 2 | 1 |  |  |  |  |  |  |  |  | ≤0.03 | ≤0.03 | - | - | - |
| Penicillin G |  | 23 | 112 | 67 | 7 | 7 | 9 | 3 | 3 |  |  | 1 |  |  |  |  |  |  | 0.03 | 0.12 | 93.1 | 3.9 | 3.0 |
| Penicillin/streptomycin^**^ | 1 | 21 | 108 | 71 | 8 | 6 | 9 | 7 | 1 |  |  |  |  |  |  |  |  |  | 0.03 | 0.12 | - | - | - |
| Cefquinome |  | 11 | 116 | 76 | 19 | 3 | 1 | 1 | 1 | 4 |  |  |  |  |  |  |  |  | 0.03 | 0.12 | - | - | - |
| Ceftiofur |  |  |  | 5 | 78 | 101 | 34 | 5 | 3 | 2 | 2 | 2 |  |  |  |  |  |  | 0.25 | 0.5 | 97.4 | 0.9 | 1.7 |
| Colistin |  |  |  |  |  |  |  |  |  |  |  |  | 2 | 9 | 221 |  |  |  | >64 | >64 | - | - | - |
| Danofloxacin |  |  |  |  | 3 | 33 | 140 | 49 | 4 | 1 | 1 | 1 |  |  |  |  |  |  | 0.5 | 1 | - | - | - |
| Enrofloxacin |  |  |  | 1 | 17 | 106 | 95 | 9 | 1 | 2 |  | 1 |  |  |  |  |  |  | 0.25 | 0.5 | 94.4 | 3.9 | 1.7 |
| Marbofloxacin |  |  |  |  |  | 7 | 99 | 117 | 6 | 1 | 1 |  | 1 |  |  |  |  |  | 1 | 1 | - | - | - |
| Florfenicol |  |  |  |  |  |  |  | 14 | 201 | 17 |  |  |  |  |  |  |  |  | 2 | 2 | 92.7 | 7.3 | 0.0 |
| Thiamphenicol |  |  |  |  |  |  |  |  | 26 | 197 | 9 |  |  |  |  |  |  |  | 4 | 4 | - | - | - |
| Doxycycline |  |  |  | 6 | 38 | 14 | 3 | 2 | 2 | 2 | 70 | 94 | 1 |  |  |  |  |  | 8 | 16 | - | - | - |
| Tetracycline |  |  |  |  |  | 7 | 4 | 28 | 12 | 7 | 6 | 12 | 28 | 121 | 7 |  |  |  | 64 | 64 | 4.7 | 12.1 | 83.2 |
| Gamithromycin |  |  |  | 34 | 49 | 3 | 1 | 3 | 3 |  | 3 |  | 5 | 36 | 23 | 72 |  |  | 64 | >128 | - | - | - |
| Tulathromycin |  |  |  |  | 1 | 2 | 6 | 57 | 21 | 2 | 5 | 1 | 4 | 4 |  | 18 | 111 |  | 256 | >256 | - | - | - |
| Tildipirosin |  |  |  |  |  |  |  |  | 7 | 7 | 66 | 8 | 3 | 1 | 1 | 139 |  |  | >256 | >256 | - | - | - |
| Tilmicosin |  |  |  |  | 2 |  |  |  | 3 | 68 | 16 | 7 |  | 5 | 1 | 130 |  |  | >128 | >128 | - | - | - |
| Tylosin |  |  |  |  | 1 | 1 | 16 | 70 | 5 | 1 |  |  |  |  | 1 | 4 | 133 |  | >256 | >256 | - | - | - |
| Tiamulin |  |  |  |  | 6 | 12 | 16 | 93 | 75 | 9 | 3 | 5 | 6 | 4 | 3 |  |  |  | 1 | 4 | - | - | - |
| Lincomycin |  |  |  |  |  |  |  |  |  | 91 |  | 3 | 2 |  | 9 | 127 |  |  | >128 | >128 | - | - | - |
| Lincomycin/spectinomycin |  |  |  |  |  |  |  | 73 | 18 | 27 | 70 | 27 | 2 |  | 7 | 5 | 2 | 1 | 4 | 16 | - | - | - |
| Spectinomycin |  |  |  |  |  |  |  |  |  | 1 | 17 | 98 | 91 | 9 | 1 | 1 | 7 | 7 | 16 | 64 | - | - | - |
| Trimetho/sulfamethoxazole^***^ | 1 | 2 | 6 | 57 | 57 | 44 | 28 | 12 | 7 | 9 | 5 |  | 4 |  |  |  |  |  | 0.12 | 2 | - | - | - |

The dilution ranges tested are those contained in the white area. Values above this range indicate MIC values higher than the highest concentration within the range. Values corresponding to the lowest concentration tested indicate MIC values lower or equal to the lowest concentration within the range. When available, susceptible and resistance breakpoints are indicated in vertical green and red lines. A dash indicates that no figure could be calculated because no CLSI interpretive criteria are available.

*Concentration for amoxicillin given, tested with clavulanic acid in a concentration ratio 2:1.

**Concentration of penicillin is given, tested with streptomycin in a concentration ratio 1:2.

***Concentration for trimethoprim given, tested with sulfamethoxazole in a concentration ratio 1:19.

|  |  |  |  |  |  |  |  |  |  |  |
| --- | --- | --- | --- | --- | --- | --- | --- | --- | --- | --- |

**Table S10**

MIC distribution frequencies of *Bordetella bronchiseptica* pig isolates from the EU 2019 – 2020.

|  | *B. bronchiseptica* (*n* = 90) | | | | | | | | | | | | | | | | | | | | | | | | |
| --- | --- | --- | --- | --- | --- | --- | --- | --- | --- | --- | --- | --- | --- | --- | --- | --- | --- | --- | --- | --- | --- | --- | --- | --- | --- |
| Antimicrobial Agent | MIC values (µg ml^-1^) | | | | | | | | | | | | | | | | | | |  | MIC_50_  (µg ml^-1^) | MIC_90_  (µg ml^-1^) | S  (%) | I  (%) | R  (%) |
|  | 0.002 | 0.004 | 0.008 | 0.015 | 0.03 | 0.06 | 0.12 | 0.25 | 0.5 | 1 | 2 | 4 | 8 | 16 | 32 | 64 | 128 | 256 | 512 | >512 |  |  |  |  |  |
| Amoxicillin |  |  |  |  |  |  |  |  |  |  |  |  | 18 | 38 | 26 | 6 | 2 |  |  |  | 16 | 32 | 0.0 | 0.0 | 100.0 |
| Amoxicillin/clavulanic acid^*^ |  |  |  |  |  |  |  |  |  |  | 6 | 71 | 7 | 5 | 1 |  |  |  |  |  | 4 | 8 | - | - | - |
| Penicillin G |  |  |  |  |  |  |  |  |  |  |  |  |  | 1 | 89 |  |  |  |  |  | >16 | >16 | - | - | - |
| Penicillin/streptomycin^**^ |  |  |  |  |  |  |  |  |  |  |  |  |  |  | 90 |  |  |  |  |  | >16 | >16 | - | - | - |
| Cefquinome |  |  |  |  |  |  |  |  |  |  |  | 1 |  | 89 |  |  |  |  |  |  | >8 | >8 | - | - | - |
| Ceftiofur |  |  |  |  |  |  |  |  |  |  |  |  | 1 | 89 |  |  |  |  |  |  | >8 | >16 | - | - | - |
| Colistin |  |  |  |  |  | 3 | 81 | 4 |  | 2 |  |  |  |  |  |  |  |  |  |  | 0.12 | 0.12 |  |  |  |
| Danofloxacin |  |  |  |  |  |  |  |  | 8 | 76 | 4 | 1 | 1 |  |  |  |  |  |  |  | 1 | 1 | - | - | - |
| Enrofloxacin |  |  |  |  |  |  |  | 1 | 71 | 12 | 4 | 2 |  |  |  |  |  |  |  |  | 0.5 | 1 | - | - | - |
| Marbofloxacin |  |  |  |  |  |  |  | 1 | 73 | 10 | 5 | 1 |  |  |  |  |  |  |  |  | 0.5 | 1 | - | - | - |
| Florfenicol |  |  |  |  |  |  |  |  |  |  |  | 44 | 39 | 1 | 3 | 3 |  |  |  |  | 8 | 8 | 0.0 | 48.9 | 51.1 |
| Thiamphenicol |  |  |  |  |  |  |  |  |  |  |  |  | 2 | 7 | 30 | 51 |  |  |  |  | >32 | >32 | - | - | - |
| Doxycycline |  |  |  |  |  | 6 | 51 | 8 | 13 | 5 |  | 2 | 4 | 1 |  |  |  |  |  |  | 0.12 | 1 | - | - | - |
| Tetracycline |  |  |  |  |  |  |  | 19 | 42 | 7 | 14 |  |  | 3 |  | 2 | 3 |  |  |  | 0.5 | 2 | - | - | - |
| Gamithromycin |  |  |  |  |  |  |  |  |  | 2 | 41 | 41 | 5 | 1 |  |  |  |  |  |  | 4 | 4 | - | - | - |
| Tulathromycin |  |  |  |  |  |  |  |  |  |  | 10 | 61 | 18 |  | 1 |  |  |  |  |  | 4 | 8 | 98.9 | 1.1 | 0.0 |
| Tildipirosin |  |  |  |  |  |  |  |  |  |  | 1 | 23 | 59 | 7 |  |  |  |  |  |  | 8 | 8 | 92.2^†^ | - | - |
| Tilmicosin |  |  |  |  |  |  |  |  |  |  |  |  |  | 5 | 69 | 16 |  |  |  |  | 32 | 64 | - | - | - |
| Tylosin |  |  |  |  |  |  |  |  |  |  |  |  |  |  |  |  | 6 | 54 | 30 |  | 256 | >256 | - | - | - |
| Tiamulin |  |  |  |  |  |  |  |  |  |  |  |  |  |  | 1 | 18 | 39 | 32 |  |  | 128 | >128 | - | - | - |
| Lincomycin |  |  |  |  |  |  |  |  |  | |  |  |  |  |  |  | 1 | 89 |  |  | >128 | >128 | - | - | - |
| Lincomycin/spectinomycin |  |  |  |  |  |  |  |  |  | |  |  |  |  |  |  |  | 3 | 79 | 8 | 512 | 512 | - | - | - |
| Spectinomycin |  |  |  |  |  |  |  |  |  | |  |  |  |  |  |  |  |  | 2 | 88 | >512 | >512 | - | - | - |
| Trimetho/sulfamethoxazole*** |  |  |  | 2 | 9 | 13 | 5 | 1 |  | 3 | 1 | 6 | 38 | 10 | 2 |  |  |  |  |  | 8 | 16 | - | - | - |

The dilution ranges tested are those contained in the white area. Values above this range indicate MIC values higher than the highest concentration within the range. Values corresponding to the lowest concentration tested indicate MIC values lower or equal to the lowest concentration within the range. When available, susceptible and resistance breakpoints are indicated in vertical green and red lines. A dash indicates that no figure could be calculated because no CLSI interpretive criteria are available.

^†^Eight isolates non-susceptible to tildipirosin.

*Concentration for amoxicillin given, tested with clavulanic acid in a concentration ratio 2:1.

**Concentration of penicillin is given, tested with streptomycin in a concentration ratio 1:2.

***Concentration for trimethoprim given, tested with sulfamethoxazole in a concentration ratio 1:19.

**Table S11**

MIC distribution frequencies of *Glaesserella parasuis* pig isolates from the EU 2019 – 2020.

| *G. parasuis* (*n* = 37) | | | | | | | | | | | | | | | | | | | | | | | | |
| --- | --- | --- | --- | --- | --- | --- | --- | --- | --- | --- | --- | --- | --- | --- | --- | --- | --- | --- | --- | --- | --- | --- | --- | --- |
| Antimicrobial Agent | MIC values (µg ml^-1^) | | | | | | | | | | | | | | | | | | | MIC_50_  (µg ml^-1^) | MIC_90_  (µg ml^-1^) | S  (%) | I  (%) | R  (%) |
|  | 0.002 | 0.004 | 0.008 | 0.015 | 0.03 | 0.06 | 0.12 | 0.25 | 0.5 | 1 | 2 | 4 | 8 | 16 | 32 | 64 | 128 | 256 | 512 |  |  |  |  |  |
| Amoxicillin |  | 9 |  |  | 1 | 6 | 7 | 3 | 3 | 2 | 1 | 1 | 2 | 1 |  |  | 1 |  |  | 0.12 | 8 | - | - | - |
| Amoxicillin/clavulanic acid^*^ |  |  |  |  | 9 | 7 | 4 | 7 | 3 | 5 | 1 |  | 1 |  |  |  |  |  |  | 0.12 | 1 | - | - | - |
| Penicillin G |  | 11 |  |  | 3 | 4 | 9 | 6 |  |  |  |  | 1 | 1 | 2 |  |  |  |  | 0.06 | 8 | - | - | - |
| Penicillin/streptomycin^**^ |  | 11 |  |  | 3 | 4 | 8 | 7 |  |  |  |  | 1 |  | 3 |  |  |  |  | 0.06 | 8 | - | - | - |
| Cefquinome |  | 4 | 2 | 6 | 5 | 5 | 2 | 5 | 3 |  | 4 |  | 1 |  |  |  |  |  |  | 0.06 | 2 | - | - | - |
| Ceftiofur | 12 | 6 | 1 | 1 | 1 |  | 7 | 3 | 1 | 1 | 1 | 1 | 1 | 1 |  |  |  |  |  | 0.008 | 2 | - | - | - |
| Colistin |  |  |  |  |  | 20 | 15 |  |  | 1 | 1 |  |  |  |  |  |  |  |  | ≤0.06 | 0.12 | - | - | - |
| Danofloxacin |  | 11 | 6 | 11 | 4 |  | 2 |  |  |  | 2 | 1 |  |  |  |  |  |  |  | 0.015 | 0.12 | - | - | - |
| Enrofloxacin |  | 14 | 10 | 5 | 3 | 1 | 1 |  |  | 1 | 1 | 1 |  |  |  |  |  |  |  | 0.008 | 0.12 | - | - | - |
| Marbofloxacin |  | 11 | 4 | 13 | 3 | 2 | 1 |  |  | 1 | 2 |  |  |  |  |  |  |  |  | 0.015 | 1 | - | - | - |
| Florfenicol |  |  |  |  |  |  | 11 | 12 | 7 | 3 | 1 | 1 |  |  | 2 |  |  |  |  | 0.25 | 4 | - | - | - |
| Thiamphenicol |  |  |  |  |  |  | 12 | 4 | 10 | 7 | 2 |  |  |  |  | 2 |  |  |  | 0.5 | 2 | - | - | - |
| Doxycycline |  |  |  |  |  | 16 | 4 | 6 | 6 | 2 | 1 | 1 |  |  | 1 |  |  |  |  | 0.12 | 1 | - | - | - |
| Tetracycline |  |  |  |  |  | 13 | 3 | 8 | 6 | 2 | 1 |  | 1 |  | 2 | 1 |  |  |  | 0.25 | 8 | - | - | - |
| Gamithromycin |  |  |  |  |  | 16 | 6 | 5 | 5 | 2 | 3 |  |  |  |  |  |  |  |  | 0.12 | 1 | - | - | - |
| Tulathromycin |  |  |  |  |  |  | 12 | 3 | 3 | 5 | 5 | 5 | 3 |  | 1 |  |  |  |  | 1 | 8 | - | - | - |
| Tildipirosin |  |  |  | 9 | 1 | 2 | 1 | 3 | 7 | 2 | 6 | 3 | 1 | 1 | 1 |  |  |  |  | 0.5 | 4 | - | - | - |
| Tilmicosin |  |  |  |  |  |  | 13 | 3 | 3 | 5 | 7 | 1 | 3 | 1 | 1 |  |  |  |  | 0.5 | 8 | - | - | - |
| Tylosin |  |  |  |  |  |  | 8 | 3 | 1 | 1 | 6 | 3 | 7 | 2 | 4 |  | 2 |  |  | 2 | 32 | - | - | - |
| Tiamulin |  |  |  |  |  |  | 10 | 2 | 4 | 5 | 6 | 4 | 6 |  |  |  |  |  |  | 1 | 8 | - | - | - |
| Lincomycin |  |  |  |  |  |  |  |  |  |  |  | 25 | 7 | 2 | 1 |  | 1 | 1 |  | ≤4 | 16 | - | - | - |
| Lincomycin/spectinomycin |  |  |  |  |  |  |  |  |  | 23 | 6 | 5 |  |  | 1 |  | 1 | 1 |  | ≤1 | 4 | - | - | - |
| Spectinomycin |  |  |  |  |  |  |  |  |  |  | 17 | 8 | 4 | 4 | 1 |  |  | 1 | 2 | 4 | 32 | - | - | - |
| Trimetho/sulfamethoxazole^***^ |  | 10 | 2 |  |  | 2 | 2 | 3 | 4 | 4 | 1 | 3 | 1 | 2 | 3 |  |  |  |  | 0.25 | 16 | - | - | - |

The dilution ranges tested are those contained in the white area. Values above this range indicate MIC values higher than the highest concentration within the range. Values corresponding to the lowest concentration tested indicate MIC values lower or equal to the lowest concentration within the range.

*Concentration for amoxicillin given, tested with clavulanic acid in a concentration ratio 2:1.

**Concentration of penicillin is given, tested with streptomycin in a concentration ratio 1:2.

***Concentration for trimethoprim given, tested with sulfamethoxazole in a concentration ratio 1:19.
